# Automated Diagnosis of Various Gastrointestinal Lesions Using a Deep Learning–Based Classification and Retrieval Framework With a Large Endoscopic Database: Model Development and Validation

**DOI:** 10.2196/18563

**Published:** 2020-11-26

**Authors:** Muhammad Owais, Muhammad Arsalan, Tahir Mahmood, Jin Kyu Kang, Kang Ryoung Park

**Affiliations:** 1 Division of Electronics and Electrical Engineering Dongguk University Seoul Republic of Korea

**Keywords:** artificial intelligence, endoscopic video retrieval, content-based medical image retrieval, polyp detection, deep learning, computer-aided diagnosis

## Abstract

**Background:**

The early diagnosis of various gastrointestinal diseases can lead to effective treatment and reduce the risk of many life-threatening conditions. Unfortunately, various small gastrointestinal lesions are undetectable during early-stage examination by medical experts. In previous studies, various deep learning–based computer-aided diagnosis tools have been used to make a significant contribution to the effective diagnosis and treatment of gastrointestinal diseases. However, most of these methods were designed to detect a limited number of gastrointestinal diseases, such as polyps, tumors, or cancers, in a specific part of the human gastrointestinal tract.

**Objective:**

This study aimed to develop a comprehensive computer-aided diagnosis tool to assist medical experts in diagnosing various types of gastrointestinal diseases.

**Methods:**

Our proposed framework comprises a deep learning–based classification network followed by a retrieval method. In the first step, the classification network predicts the disease type for the current medical condition. Then, the retrieval part of the framework shows the relevant cases (endoscopic images) from the previous database. These past cases help the medical expert validate the current computer prediction subjectively, which ultimately results in better diagnosis and treatment.

**Results:**

All the experiments were performed using 2 endoscopic data sets with a total of 52,471 frames and 37 different classes. The optimal performances obtained by our proposed method in accuracy, F1 score, mean average precision, and mean average recall were 96.19%, 96.99%, 98.18%, and 95.86%, respectively. The overall performance of our proposed diagnostic framework substantially outperformed state-of-the-art methods.

**Conclusions:**

This study provides a comprehensive computer-aided diagnosis framework for identifying various types of gastrointestinal diseases. The results show the superiority of our proposed method over various other recent methods and illustrate its potential for clinical diagnosis and treatment. Our proposed network can be applicable to other classification domains in medical imaging, such as computed tomography scans, magnetic resonance imaging, and ultrasound sequences.

## Introduction

Various types of gastrointestinal (GI) disorders, such as a tumors, ulcerative colitis, irritable bowel syndrome, hemorrhoids, *Helicobacter pylori*, Crohn disease, polyps, and colorectal cancer, are among the leading causes of death [1]. In the United States, about 76,940 people died in 2016 due to different types of gastric cancers, according to the American Cancer Society [1]. Early and accurate diagnosis of severe diseases, such as polyps or tumors, using endoscopy videos is of great significance and leads to better treatment. However, the subjective diagnosis of such GI diseases is not only a tedious and time-consuming task but also requires sufficient knowledge and clinical experience. These diagnostic problems can be solved to a great extent by developing effective computer-aided diagnosis (CAD) tools that provide a fully automated way of detecting and classifying different GI diseases. CAD tools can assist medical experts in effective diagnosis and treatment during the initial stage of severe medical conditions [2-10]. Figure S1 in Multimedia Appendix 1 presents an overall workflow diagram of a CAD tool to visualize its clinical usability and significance in making a diagnostic decision. In the first step, medical professionals use a particular type of imaging modality to visualize the internal structure of body organs, such as the GI tract. After that, a CAD model analyzes the visual data (obtained in the first step) to highlight the lesions or suspicious regions. Finally, these highlighted results further assist the medical experts in making an effective diagnostic decision in a short time.

In the last few years, deep learning algorithms, especially artificial neural networks (ANNs), have significantly enhanced the performance of artificial intelligence–based CAD tools, which are used for diagnostic purposes in various medical domains [11-15]. In general, these ANN models undergo a training procedure to learn the optimal representation of the training data set [16] by using optimization algorithms, such as stochastic gradient descent [17]. In this way, a deep learning–based trained model that contains the optimal representation of the training data set in trainable parameters is obtained. In clinical practice, this model can analyze newly acquired endoscopic videos or images by using previous knowledge of trainable parameters. Various types of ANN models have been proposed in the image analysis domain. Among these models, convolutional neural networks (CNNs) [16] have gained special attention due to their superior performance in various image recognition–based applications, including in medical fields. The convolutional layers are considered the key part of a CNN model and contain trainable filters of different depths and sizes. These filters are trained during the training procedure by extracting complex hidden patterns (also known as deep features) from the training data set.

Over the past few years, considerable contributions have been made by robust and efficient CAD tools in the endoscopy domain. However, most of these methods are designed to detect specific types of GI diseases, such as polyps, ulcers, tumors, or cancer, using handcrafted or deep features–based approaches. Before the advent of deep features–based methods, most studies used handcrafted features such as color and texture information to perform the automated detection and classification of particular types of GI disease [18-24]. In recent years, various deep learning–based CAD tools have been proposed for endoscopic video and image analysis [2-10]. Such deep learning–based CAD tools are capable of performing the classification and detection of different GI abnormalities in a more precise and accurate way than the previous handcrafted features–based methods. However, most of the previous deep learning–based CAD methods used only the spatial information for the automatic diagnosis of GI diseases, which reduced the overall diagnostic performance. The internal structure of the human GI tract is captured as a moving sequence (video) with respect to time during an endoscopy procedure. Therefore, an endoscopic video encompasses both spatial and temporal information. In a video, the temporal information exists in the sequence of consecutive frames and provides essential information. Therefore, it is possible to use both spatial and temporal information in developing a high-performance CAD tool with good diagnostic capability. A comprehensive analysis of existing studies [2-10,18-24] in comparison with our proposed method can be found in Multimedia Appendix 1.

The motivation behind of this study was the development of a comprehensive CAD framework that would be able to recognize a wide range of GI diseases simultaneously rather than multiple CAD tools used one by one to detect an anonymous GI disease. To accomplish this task, we considered a total of 37 different classes (including both diseased and normal cases) related to the human GI tract, which is significantly more than most recent studies. Another motivation was the included cross-validation mechanism in the proposed CAD tool that provides visual information about its diagnostic decision. Such additional information can assist medical experts in validating the computer decision interactively. Therefore, in this research work, we use the strength of recent AI techniques in the endoscopy domain and propose a high-performance classification and retrieval framework for multiple GI diseases using endoscopic videos. Mainly, the overall pipeline of the proposed classification network is composed of a densely connected convolutional network (DenseNet), our defined long short-term memory (LSTM) network using LSTM cells, principal component analysis (PCA), and the k-nearest neighbors (KNN) algorithm. Experimental results demonstrate the superiority of the proposed CAD framework in comparison with various state-of-the-art methods. This study provides five main contributions.

First, this is the first spatiotemporal feature–based CAD framework based on the integrated DenseNet and LSTM followed by a PCA-based KNN classifier for the effective diagnosis of various GI diseases.

Second, with the addition of PCA, our method reduces the feature dimension up to 95%, with the gain of an average accuracy of 3.62% in comparison with previous work [10].

Third, we include the retrieval framework after the classification network to validate the CAD decision subjectively.

Fourth, in our framework, the number of successive frames to be classified can be variable rather than using the fixed-length sequence.

Fifth, we have made our trained model publicly available through Dongguk University [25], along with the information regarding training and testing data splitting.

## Methods

### Study Design

In this section, a detailed description of our proposed method is formulated in sequential order. An overall description of a class prediction–based video or image retrieval system using our proposed spatiotemporal feature–based classification network is given, followed by a detailed explanation of the proposed classification network, which includes spatiotemporal feature extraction and classification stages.

### An Overview of the Proposed Approach

**Figure 1:**
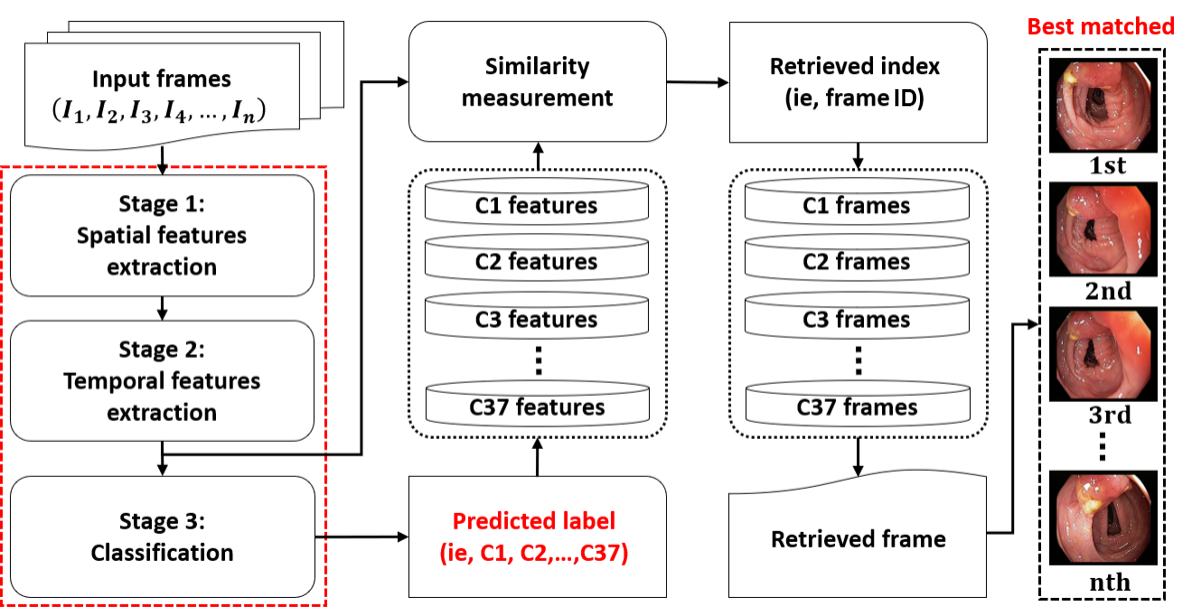
Comprehensive flow diagram of the proposed classification and retrieval framework. The red dotted box highlights our major contributions in this proposed retrieval framework.

In general image or video classification and retrieval frameworks, the key element is the optimal representation of visual information or features. The optimal features are further used in retrieving relevant information from the image or video database based on feature-matching mechanisms, such as the minimum Euclidian distance. Thus, the overall performance of such a system is directly related to the methods that perform the optimal feature extraction task. Recently, deep learning–based feature extraction methods have shown the best performance in various image- and video-processing domains. Therefore, in our proposed framework, the strength of such deep learning–based algorithms was used to obtain high classification and high retrieval performance in endoscopy. A comprehensive flow diagram of our proposed framework is shown in Figure 1.

First, deep feature–based spatial and temporal information was extracted from the given input endoscopy video of *n* successive frames by using a cascaded DenseNet and LSTM-based network consecutively. In this way, a collective spatiotemporal feature vector was obtained for the given endoscopy video, which was further used at the classification stage (after applying the PCA [26]) to predict the class label. Second, the predicted class label was used to select the set of relevant class features from the feature database, and these were further used at the feature matching stage. Next, the extracted spatiotemporal feature vector (obtained in the first step from the input query sequence) was matched with the set of selected features (obtained in the second step), and retrieval information (ie, frame ID) was obtained based on the best-matching results. Finally, class prediction–based retrieval was done by selecting the best-matched cases from the entire database based on retrieval information.

### The Structure of Our Proposed Spatiotemporal Feature–Based Classification Model

**Figure 2:**
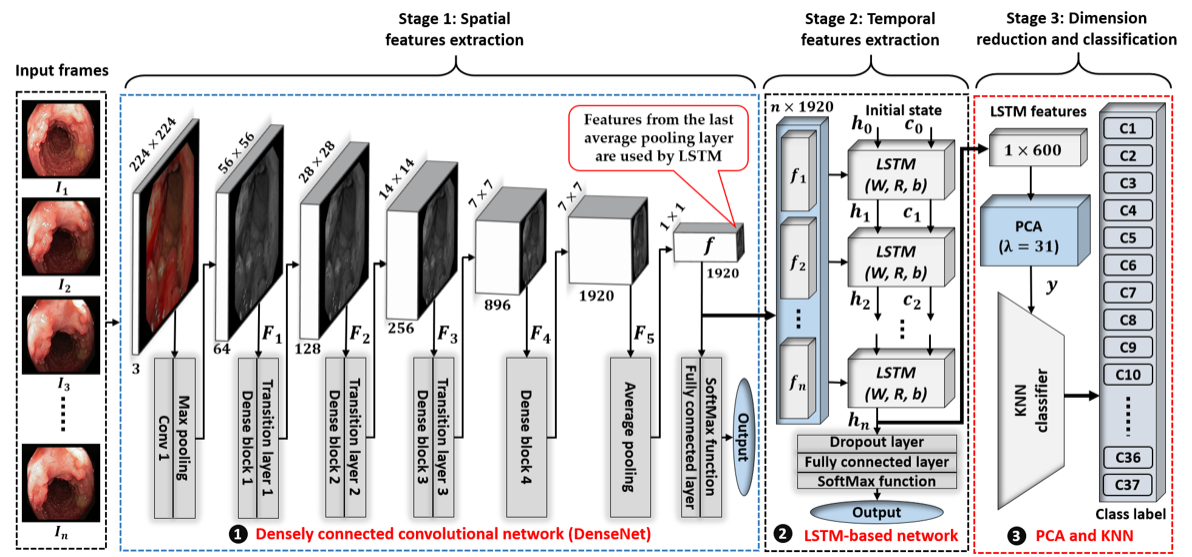
Overall block diagram of our proposed spatiotemporal feature–based classification network composed of DenseNet and LSTM-based networks. KNN: k-nearest neighbor; LSTM: long short-term memory; PCA: principal component analysis.

Our proposed spatiotemporal feature–based classification model is composed of a DenseNet followed by the LSTM model to extract spatial and temporal features, respectively. Figure 2 presents an overall block diagram of our proposed spatiotemporal feature–based classification model. For better understanding, the complete structure of our model is divided into three main stages. In the first stage, each frame of the given input video of *n* consecutive frames (ie, *I*_1_, *I*_2_, *I*_3_…, *I_n_*) is processed by the DenseNet to extract the spatial features. Here, the factor *n* presents the length of the input frames, which control the span of temporal features with respect to time. The total time span of *n* consecutive frames can be calculated by multiplying *n* with the frame rate, which is 30 (ie, 30 frames per second) in the case of our selected data set. Furthermore, these extracted features are processed by the LSTM-based network in the second stage to exploit the temporal information. Finally, a single feature vector is obtained corresponding to each input video sequence, which is further fed to the dimension reduction and classification stages to reduce the feature dimensions and then predict the class label, respectively. The comprehensive details of each stage are given in the succeeding sections.

#### Spatial Feature Extraction Using DenseNet

The first stage of our proposed classification framework was composed of a well-known CNN architecture called DenseNet. Its main goal was to extract the spatial features from each frame of the given input video sequence independently. The main reason for selecting a DenseNet was that its classification performance is superior to other modern deep CNN models. Moreover, the dense connectivity in our selected model required fewer parameters than an equivalent traditional CNN. It also overcomes the vanishing gradient problem due to the presence of shortcut connectivity. During network training, the trainable parameters (ie, filter weights) receive an update proportional to the gradient value. In the case of the vanishing gradient problem, the gradient value becomes so small that it ultimately results in a small update and finally stops the training procedure. Such poor training significantly affects the overall performance of the network. However, the presence of shortcut connectivity in DenseNet overcomes this problem and results in better performance.

The complete layer-wise structure of the DenseNet is shown in Figure 2 and Table S2 in Multimedia Appendix 1. The entire network mainly comprises multiple dense blocks and transition layers, which could be considered the basic building blocks. There are a total of 4 dense blocks and 3 transition layers in it, and these make a significant contribution to exploiting discriminative features for better classification performance. The detailed structure of a single dense block followed by the transition layer is visualized in Figure S2 in Multimedia Appendix 1.

Each dense block is made up of multiple pairs of convolutional layers (conv 1 × 1 and conv 3 × 3) in sequential order. Furthermore, a feature concatenation layer is also added after each pair of convolutional layers (conv 1 × 1 and conv 3 × 3) to concatenate the output feature maps of the current pair (conv 1 × 1 and conv 3 × 3) with the previous pair within the same dense block. In this way, each subsequent pair of convolutional layers directly accesses the output of all the previous pairs within the same block. A generalized expression to evaluate the output of the ℓth pair is given as follows: 

*x*_ℓ_ = *H*_ℓ_ ([*x*_0_, *x*_1_, *x*_2_, …, *x*_ℓ__– 1_]) **(1)**

In equation 1, *H*_ℓ_ means the operation of the ℓth pair of convolutional layers (conv 1 × 1 and conv 3 × 3), which considers all the feature maps (*x*_0_, *x*_1_, *x*_2_, …, *x*_ℓ__– 1_) of the previous pairs within the same block. There is also another hyperparameter (labeled growth rate) in each dense block that regulates the increase in the depth of the output feature maps after passing through each pair of convolutional layers. Whereas the dimensions of the feature maps remain the same within each dense block, the number of filters between them changes. A generalized expression for the ℓth pair of convolutional layers (in each dense block) can be expressed as:

*k*_ℓ_ = *k*_0_ + *k* × (ℓ – 1) **(2)**

In equation 2, *k* is the growth rate of the network; this hyperparameter was 32 in the original DenseNet model [27]. *k*_0_ is the initial depth of the input feature map, *x*_0_, that was passed to the dense block, and *k*_ℓ_ is the output depth of the ℓth pair of convolutional layers. Finally, the transition layer further processes the output feature map *x*_ℓ_ of the dense block and reduces its depth and dimension by passing it through convolutional and average pooling layers sized 1 × 1 and 2 × 2 pixels, respectively.

The given structural and parametric details (Table S2 in Multimedia Appendix 1) further illustrate the flow of spatial feature extraction through the different layers of the network. Initially, the first input layer (labeled “Image Input”) in the DenseNet is used to pass the given input image to the network for further processing. After the input layer, the first convolutional layer (labeled “Conv1”) exploits the input frame by applying a total of 64 different filters sized 7 × 7 × 3 pixels. The output feature map (generated by Conv1) is then processed by a max-pooling layer, which generates a down-sampled feature map, *F*_1_, sized 56 × 56 × 64 pixels. After the max-pooling layer, the first stack of the dense block and transition layer (labeled “Dense Block 1” and “Transition Layer 1”) processes the feature map *F*_1_ and generates a down-sampled feature map, *F*_2_, sized 28 × 28 × 128 pixels. The output feature map *F*_2_ is further processed by the second stack of the dense block and transition layer (labeled “Dense Block 2” and “Transition Layer 2”), and an output feature map, *F*_3_, sized 14 × 14 × 125 pixels is obtained. Similarly, the third stack of the dense block and transition layer (labeled “Dense Block 3” and “Transition Layer 3”) also processes the feature map *F*_3_ and generates an output feature map, *F*_4_, sized 7 × 7 × 896 pixels. Output feature map *F*_4_ is further processed by the last dense block (labeled “Dense Block 4”) and produces an output feature map, *F*_5_, sized 7 × 7 × 1920 pixels. Finally, a spatial feature vector *f* sized 1 × 1 × 1920 pixels is obtained after applying the last average pooling layer (labeled “Avg Pooling”) with a filter size of 7 × 7 pixels over the last output feature map, *F*_5_. The same procedure is repeated for all the other input frames, which ultimately generates a set of *n* feature vectors (*f*_1_, *f*_2_, *f*_3_ …, *f_n_*) for all the successive endoscopic images (*I*_1_, *I*_2_, *I*_3_ …, *I_n_*). Finally, all these feature vectors are further processed by the second-stage network for temporal feature extraction. There are also 3 other layers (labeled “fully connected,” “softmax,” and “classification output”) after the last average pooling layer, as shown in Figure 2. These 3 layers only take part in the spatial training procedure of the DenseNet. Therefore, after completing the training phase, the final spatial features are selected from the last average pooling layer and are further processed in subsequent stages.

#### Temporal Feature Extraction Using an LSTM Network

In this second stage, an LSTM-based neural network (a version of a recurrent neural network) [28] is used to learn the temporal features from the spatial features (extracted in the first stage). In the case of temporal information-based challenges, the LSTM-based neural networks overcome the vanishing gradient problem, which causes poor training of a network due to a small gradient value. This vanishing gradient problem occurs through the repeated use of a recurrent weight matrix in a recurrent neural network. However, this problem is resolved in LSTM by replacing the recurrent matrix with the identity function. Therefore, in our proposed classification framework, a simplified structure of an LSTM-based network is adopted to further enhance classification performance by extracting the temporal features. A complete network structure and layer-wise description, including the parametric details, are given in Figure 2 (stage 2) and Table S2 in Multimedia Appendix 1, respectively. In Figure 2, the LSTM block presents a standard LSTM cell, which can be considered the main building block of our proposed LSTM-based network. In a standard LSTM cell, 3 different types of learnable parameters (recurrent weights *R*, input weights *W*, and bias *b*) are involved, which are trained using the training data set. These learnable parameters (*W*, *R*, *b*) are responsible for learning the temporal features from the given training data set. Complete details about the internal structure of the LSTM cell are provided in Hochreiter and Schmidhuber [29]. For a better understanding, an unrolled version of the LSTM cell is shown in the second-stage network of Figure 2, which presents *n* executions of a single LSTM cell. Here, the parameter *n* (number of executions of a single LSTM cell) is a variable directly related to the length of a given input sequence, which provides the flexibility to classify input sequences with different numbers of frames (*I*_1_, *I*_2_, *I*_3_ …, *I_n_*).

The extracted set of *n* spatial features (*f*_1_, *f*_2_, *f*_3_ …, *f_n_*) in the previous stage is processed by this second stage network in sequential order, which can be visualized in Figure 2 (stage 2). A sequence input layer (Table S2 in Multimedia Appendix 1) is used to pass these spatial features to the LSTM layer, which is composed of multiple LSTM cells with different input parameters. In an actual scenario, a single LSTM cell is repeated *n* times to process the set of *n* spatial features (*f*_1_, *f*_2_, *f*_3_ …, *f_n_*) in sequential order using the state information (hidden state *h_n_*_– 1_ and cell state *c_n – 1_*) of all the previous input feature vectors (*f*_1_ to *f_n_*_– 1_). The hidden state *h_n_*_– 1_ holds the output of the LSTM cell for the input feature *f_n_*_– 1_, and the cell state *c_n – 1_* keeps the information learned from all the previous input feature vectors, *f*_1_ to *f_n_*_– 1_. In the case of the first input feature vector, *f*_1_, the LSTM cell considers the initial state of the network to be null values (*h*_0_ = [], *c*_0_ = []) when computing the first updated cell state *c*_1_ and output *h*_1_. For all the succeeding input feature vectors (*n*≠1), the LSTM cell considers the current state of the network (*h_n_*_– 1_, *c_n_*_–__1_) to compute the output *h_n_* and the updated cell state *c_n_*. This way, after processing all the input feature vectors (*f*_1_, *f*_2_, *f*_3_ …, *f_n_*), the last hidden state *h_n_* of the network is considered to be the final output feature vector for performing further dimension reduction and classification. There are also 4 other layers (labeled dropout, fully connected, softmax, and classification output) after the LSTM layer, as mentioned in Table S2 in Multimedia Appendix 1. These 4 layers only take part in the training procedure for this second stage network. Therefore, after training, the final features are selected from the LSTM layer for further processing in the next stage.

#### Dimension Reduction and Classification

Because the last hidden state *h_n_* of the network (with a feature dimension of 1 × 600 pixels) includes the complete spatiotemporal information for all the input feature vectors (*f*_1_, *f*_2_, *f*_3_ …, *f_n_*), it was therefore selected as the final output feature vector for classification. However, before applying the classification algorithm, a PCA was performed to further reduce the dimension of the final output feature vector *h_n_* by using MATLAB R2019a (MathWorks Inc) [30]. This step was taken to reduce the feature comparison time for retrieval purposes and improve the overall classification accuracy of the proposed classification network. Therefore, an intermediate features-based data set (as shown in Figure S3 in Multimedia Appendix 1 after the LSTM-based network) was created for all the training and testing samples by extracting the output features from the last hidden state *h_n_* of the LSTM-based network. This newly obtained data set (in terms of feature vectors) was used to perform dimension reduction using PCA. The overall average performance corresponding to the different number of eigenvectors (λ=1,2,3, …,600) was evaluated to select the optimal number of eigenvectors (λ). We obtained the best average performance for λ=31. A final set of feature vectors (with a feature dimension of 1 × 31 pixels) was created for all the training and testing data sets. Figure S3 in Multimedia Appendix 1 shows the conceptual representation of all the intermediate data sets created by our proposed classification framework at different stages. In Figure S3 in Multimedia Appendix 1, *k* presents the total number of data samples in the entire data set (including both training and testing).

Finally, the KNN [31] algorithm was applied using MATLAB R2019a [30] to classify this newly obtained features-based data set after the PCA. This simple classification algorithm was selected based on its classification performance relative to other classification algorithms, such as adaptive boosting (AdaBoostM2) [32] and a multiclass support vector machine (SVM) (Multi-SVM) [33]. It predicts the class label for the given testing sample by calculating the distance to the different neighbor samples and selecting the neighbor with the minimum distance. In our case, there were a total of 37 different categories related to the human GI tract, including both normal and abnormal cases. Therefore, the KNN algorithm finds the best class prediction for the given input testing data sample by identifying the nearest neighbor (based on Euclidean distance) of the 37 different neighbors. Finally, the predicted class label is assigned to the given testing data sample, which was the ultimate objective of our proposed classification model.

## Results

### Data Set and Preprocessing

**Figure 3:**
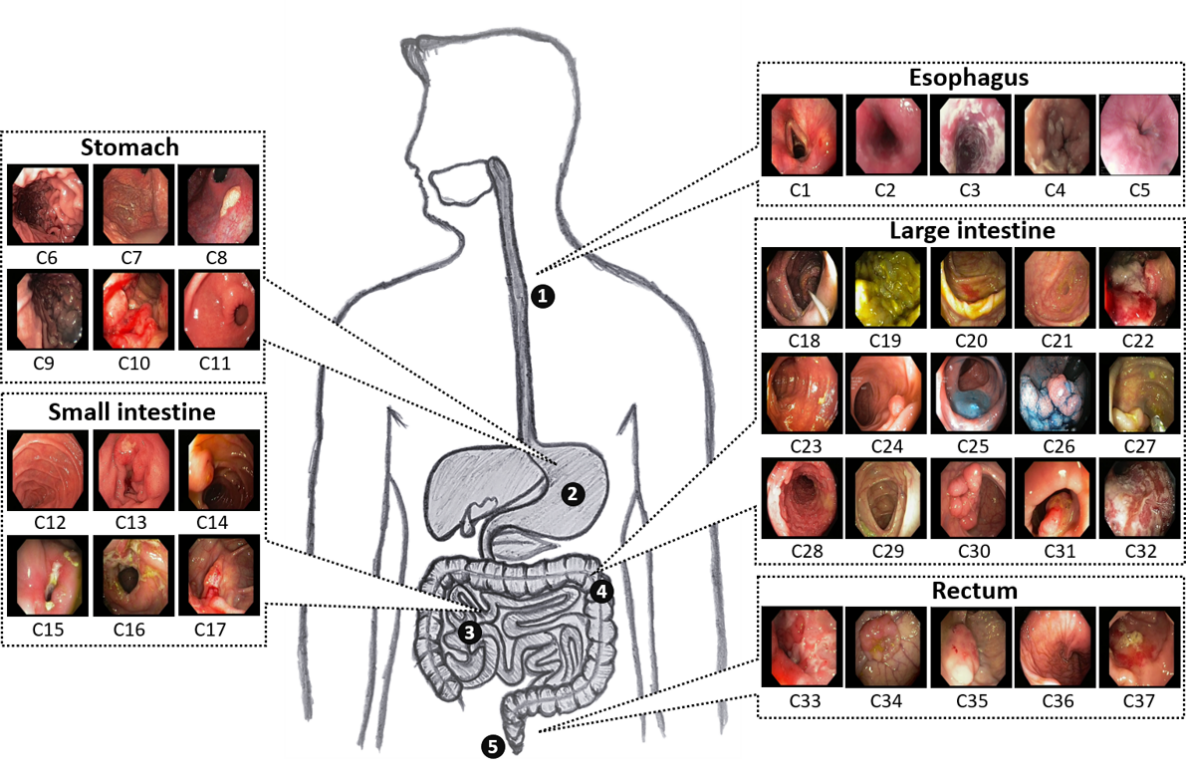
Example frames of our selected data set for each class and their correspondence with the different anatomical districts of the human gastrointestinal tract.

We evaluated the performance of our proposed classification network on 2 publicly available endoscopic databases: a dataset from Gastrolab [34] and the Kvasir dataset [35]. As the databases state [34,35], these are open databases and can be used for research purposes. These databases consist of various endoscopic videos [34] and some already extracted frames (including both normal and disease cases) [35] related to different anatomical districts of the human GI tract. From these 2 databases [34,35], we collected a total of 77 endoscopic videos with a total of 52,471 frames, as described in our previous work [10]. For each video, the anatomical districts, disease type, and other related details were provided in the video title. Based on the available information related to each video, we categorized the entire data set into 37 different classes, which encompassed the 5 main anatomical districts of the human GI tract, labeled the esophagus, the stomach, the small intestine, the large intestine, and the rectum. Figure 3 presents a pictorial representation of these different anatomical districts of the human GI tract and their corresponding groups of classes, with sample frames for each class (ie, C1 to C37). There are also other organs in the human GI tract, but the available data cover only the described 5 anatomical districts and include both normal and disease cases. Our proposed classification network shows the best performance for this data set, and it is also capable of classifying a large number of classes.

In addition, our selected data set shows high intraclass variance due to the varying textures and structures of the specific types of GI disease, such as tumors or cancer. Moreover, the dynamic structural changes during the endoscopy procedure and the different viewing conditions may also cause high intraclass variance. Figure S4 in Multimedia Appendix 1 shows a few example frames from our selected data set that illustrate this high intraclass variance. In this situation, it is difficult to capture a generalized feature-level abstraction that can represent all the possible samples of a class. However, the high intraclass variance in the data set may help analyze the performance of our proposed network in a more challenging scenario. The problem caused by the intraclass variance can be solved using a versatile and sufficient amount of training data related to each class. Such types of data may help extract a generalized high-level representation for each class by training a deep learning–based network.

Furthermore, Table S3 in Multimedia Appendix 1 provides additional details about the subcategories of each anatomical district and their corresponding classes and includes the actual class description and number of training and testing frames. Due to the different spatial resolutions, we resized all the extracted frames to a spatial dimension of 224 × 224 pixels (as the input layer size of the first-stage network) and then converted them to a BMP file format. We performed 2-fold cross-validation in all the experiments by dividing the entire data set, using 50% of the data for training and the remaining 50% for testing. The data splitting was performed by considering the first half of consecutive frames of a video as training data and the remaining half as testing data. Due to the limited size of the data set, it was not possible to use different videos in training and testing. In addition, most of the classes (about 21 classes out of 37 classes) consisted of single-patient data (ie, one video per class), as mentioned in Table S3 in Multimedia Appendix 1. Although we used the former part of a video for training and the latter for testing, the data were completely different due to the movement of the capturing device and the body organs, as shown in Figure S5 in Multimedia Appendix 1. In Figure S5 in Multimedia Appendix 1, a significant visual difference between the training and the testing data can be observed for some classes with single-patient data (ie, one video per class). Nevertheless, to highlight the superiority of our method, we also evaluated the results of some existing state-of-the-art methods [5,8,10,36-47] based on the same data set and experimental protocol. Additionally, online data augmentation [48] (with random rotation and translation in both directions) was applied (only in training the first-stage network) to resolve the class imbalance problem [49]. This class imbalance problem occurred due to the different amounts of training samples in each class; therefore, we applied online data augmentation only for the training data set.

### Experimental Setup

The proposed classification and retrieval framework was developed using a deep learning toolbox from MATLAB R2019a [30]. This toolbox provides a comprehensive framework for designing and implementing various types of ANNs, including pretrained networks. All the experiments were conducted using a standard desktop computer with a 3.50 GHz Intel Core i7-3770K central processing unit [50] and 16 GB RAM, an NVIDIA GeForce GTX 1070 graphics card [51], and the Windows 10 operating system. Both networks (stage 1 and stage 2) were trained using a well-known backpropagation algorithm called stochastic gradient descent [17]. Its main objective is to find the optimal parameters (ie, filter weights and biases) of a network iteratively by optimizing an objective function. Moreover, due to the limited data set, the initial parameters of the first-stage network were initialized with the filter weights of a pretrained CNN model (DenseNet201), which was already trained on the ImageNet data set [52]. This is a valid transfer learning approach that effectively converges the network training process, especially in the case of a small training data set. Similarly, the initial parameters of the second-stage network were assigned randomly by choosing Gaussian distribution with a mean of 0 and standard deviation of 0.001. Comprehensive details of all the training parameters are given in Table S4 in Multimedia Appendix 1.

After setting up the experimental protocol, the sequential training of the DenseNet (stage 1) and then the LSTM-based network (stage 2) was performed using the training data set. In the first stage, the DenseNet was trained using our selected data set (extracted endoscopic frames from the videos) to extract the spatial features. The progress of the training accuracy and loss according to the different numbers of epochs is visualized in Figure S6 in Multimedia Appendix 1 (for both folds of cross-validation). As seen in the figure, after increasing the number of epochs, the training accuracy approached 100% and the training loss decreased to 0, which shows that our network has been trained. In the second stage, the LSTM-based network was trained to further extract the temporal information using the spatial features–based data set (extracted from the DenseNet after completing the training). In this stage, the *n* successive feature vectors (extracted from the *n* successive frames in the first stage) were considered to be one training sample. Therefore, we converted each training sample sized 1 × 1920 pixels to *n* × 1920 pixels by embedding the previous *n* – 1 feature vectors (extracted from the *n* – 1 consecutive frames). This newly obtained data set was used to train the second-stage network to further exploit the temporal information. We considered the optimal number of successive frames for training to be 14 (ie, *n*=14) after performing a number of experiments. The subsequent sections provide a detailed explanation for the experimental results for different values of *n*. Figure S7 in Multimedia Appendix 1 visualizes the training progress (in terms of training loss and accuracy) of the LSTM-based network according to different numbers of epochs. As the figure shows, the network convergence speed is faster and smoother than DenseNet. The main reason for this is the use of a spatial features–based data set rather than the original frames in this second-stage network for further temporal feature extraction. Finally, we assessed the performance of our proposed classification network using 4 quantitative evaluation metrics: accuracy, F1 score, mean average precision (mAP), and mean average recall (mAR) [53]. These are the most common measures for calculating the overall performance of a network from all perspectives.

### Testing of the Proposed Method

The optimum number of successive frames (*n*) plays a vital role in exploiting temporal information, which ultimately results in better classification performance. A small value of *n* incorporates fewer temporal features, while a high value of *n* increases the effect of noise and the processing time. Therefore, it was necessary to find the optimal number of successive frames. For this purpose, we assessed the overall performance of our proposed classification network by considering different numbers of successive frames (ie, *n* = 1,2,3, …,20) in both the training and testing phase. The ultimate objective of these experiments was to find the value of *n* that showed the best performance. Figure S8 in Multimedia Appendix 1 shows the average performance of our proposed network for different values of *n*. We obtained the best average performance (as highlighted with the green square box in Figure S8 in Multimedia Appendix 1) for *n*=14. Therefore, we considered *n*=14 to be the optimal training parameter that would exploit temporal information while achieving significant performance gain, and we performed all the other experiments with this parameter setting.

After selecting the optimal value of *n* for the training of the LSTM-based network, the performance of our trained network (for the optimal value of *n*) was also evaluated by considering different numbers of successive frames (ie, *n* = 1,2,3, …,150). Figure S9 in Multimedia Appendix 1 shows the average classification performance for different values of *n* (ie, *n* = 1,2,3, …,150) used only in the testing stage. These results demonstrated that the overall performance of our proposed network was directly proportional to the number of successive frames (ie, n = 1,2,3, …,150) selected in the testing phase. The main reason for this is that a greater number of successive frames encompass more temporal information, which results in better classification performance. All the performance metrics showed a similar performance gain, which illustrated the significance of our proposed network compared with conventional deep CNN models. However, it was observed that the overall change in performance gain became smaller as the number of successive frames (ie, *n* = 1,2,3, …,150) increased. In this performance analysis setup, we obtained the highest performance for *n*=146 (as highlighted with a green square box in Figure S9 in Multimedia Appendix 1) instead of higher values of *n*. Therefore, we considered *n*=146 to be the testing parameter value for our selected data set. Both parameters that showed the best results for the various recent ANN methods (*n*=14 for training and *n*=146 for testing) were selected for the complete network.

To further enhance the prediction capability of the proposed network, we performed additional experiments by applying PCA followed by KNN after the LSTM-based network (as explained in the “Methods” section). In this performance analysis setup, we evaluated the PCA-based performance for the different numbers of eigenvectors (λ = 1,2,3, …600), as shown in Figure S10 in Multimedia Appendix 1. These results were computed to find the number of eigenvectors (λ) that showed the best performance. Of all the performance results, we found the maximum average performance for λ=31, as highlighted with a green square box in Figure S10 in Multimedia Appendix 1 (left side), which presents a close-up view to further magnify the performance difference. Detailed comparative results (with and without PCA) are also given in Table 1 to show the effect of PCA on the LSTM-based network. In this analysis, the comparative results without PCA are based on a fully connected network (comprising fully connected, softmax, and classification output layers after the LSTM layer). As seen in Table 1, the PCA-based classification performance was higher than the fully connected network (without PCA). Furthermore, the dimension of the PCA-based feature vectors (1 × 31 pixels) is about 20 times lower than the original feature vectors (1 × 600 pixels), which also results in better retrieval performance. Consequently, our proposed classification network (including the PCA followed by KNN after the LSTM-based network) showed superior performance in all respects.

**Table 1:** Performance comparisons of our proposed network with and without applying the PCA.

Fold	Performance without PCA^a^ (using fully connected network), %				Performance with PCA + KNN^b^ (λ=31), %			
	Accuracy	F1	mAP^c^	mAR^d^	Accuracy	F1	mAP	mAR
Fold 1	97.31	97.83	97.97	97.70	98.41	98.80	98.82	98.79
Fold 2	94.18	95.11	96.91	93.38	93.97	95.18	97.54	92.94
Average, mean (SD)	95.75 (2.21)	96.47 (1.92)	97.44 (0.75)	95.54 (3.05)	96.19 (3.13)	96.99 (2.56)	98.18 (0.90)	95.86 (4.13)

^a^PCA: principal component analysis.

^b^KNN: k-nearest neighbor.

^c^mAP: mean average precision.

^d^mAR: mean average recall.

**Figure 4:**
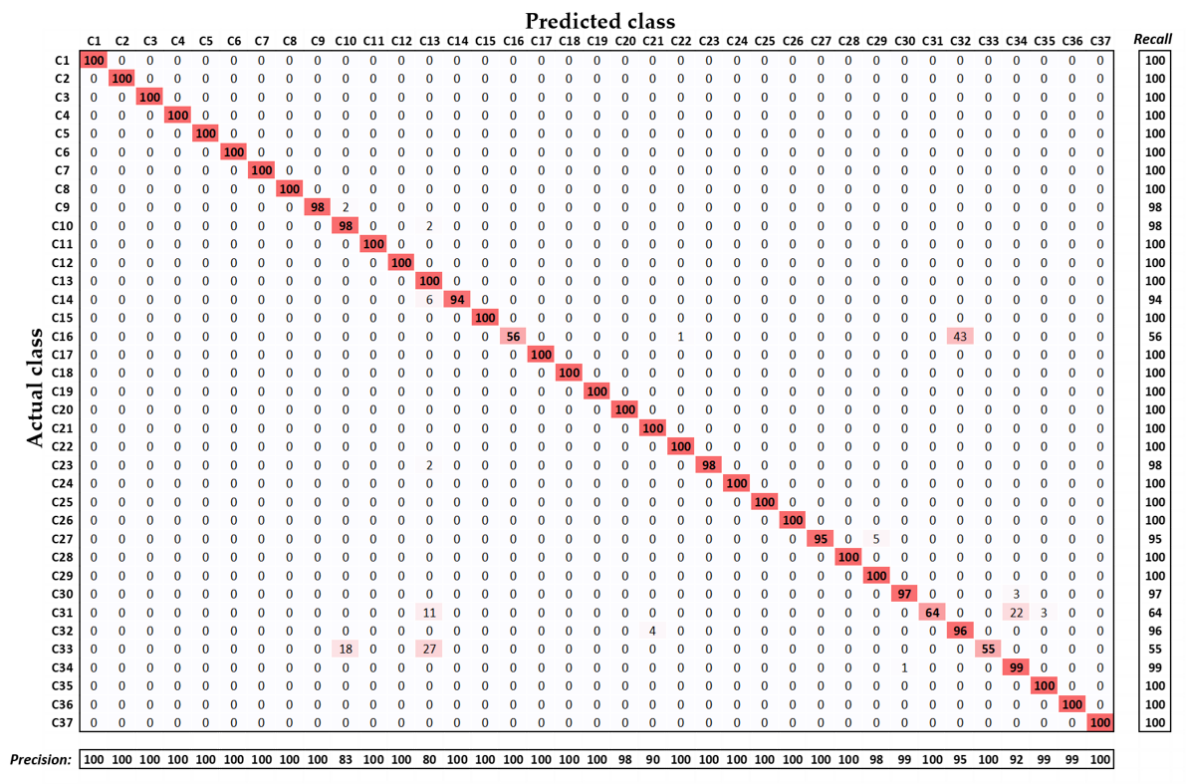
Detailed performance results of our proposed network shown in an average confusion matrix.

The detailed performance results of each class are shown in a confusion matrix in Figure 4. Each diagonal value in Figure 4 presents the individual class performance for accuracy. Moreover, the column (on the right side) and row (on the bottom) present the individual class performance for recall and precision, respectively. As seen in Figure 4, most of the classes show notably high classification performance (accuracy of at least 94%), but 3 classes show low performance (ie, C16, C31, and C33, which show average accuracies of 56%, 64%, and 55%, respectively). Such performance degradation is caused by the existence of high interclass similarity among the multiple classes. However, the overall performance of our proposed network was substantially high for a data set with high intraclass variance and high interclass similarity.

### Comparisons With Previous Methods

We conducted a detailed performance comparison of our proposed method with other state-of-the-art deep learning methods for the automated detection of different types of GI diseases [5,8,10,36-47]. To make a fair comparison, the performance of these existing baseline methods was evaluated under the same experimental setup and data set that was used for our proposed method. We evaluated the performance of a total of 15 different baseline methods to make a detailed comparison with our proposed method. In this regard, our comparative analysis was more comprehensive than those of these existing studies [5,8,10,36-47]. Table 2 provides the comparative results of all the baseline methods in comparison with our proposed method. We obtained an average performance gain of 3.62%, 3.58%, 3.60%, and 3.58% for the accuracy, F1, mAP, and mAR, respectively, compared with the second-best network [10]. Moreover, our method outperformed the original DenseNet201 [27] (third-best network, which was used in Song et al’s study [43]), with a performance gain of 4.07%, 4.57%, 5.27%, and 3.93% for the accuracy, F1, mAP, and mAR, respectively. These results signify the diagnostic ability of our proposed network for endoscopy image analysis for better treatment of various GI diseases. To envision the computational complexity of our proposed method and the baseline methods, brief parametric details of all the models are provided in Table S5 in Multimedia Appendix 1. Although the number of training parameters of our proposed network is higher than those in the second-best [10] and third-best [43] methods, its substantial performance gain distinguished it from the other models. The other baseline models [5,16,54-56] with a large number of training parameters showed lower performance compared with our proposed network.

**Table 2:** Comparative classification performance of our proposed network with the other state-of-the-art methods used in endoscopy.

Authors	Deep network	Accuracy, %	F1, %	mAP^a^, %	mAR^b^, %
Zhang et al (2017) [44]	SqueezeNet [57]	77.84	76.74	76.77	76.73
Hicks et al (2018) [45]	VGG19 [54]	85.15	85.29	85.88	84.72
Fan et al (2018) [36]	AlexNet [16]	80.08	80.49	80.70	80.28
Takiyama et al (2018) [8]	GoogLeNet [58]	84.59	85.14	85.29	84.99
Byrne et al (2019) [5]	InceptionV3 [55]	87.92	88.45	87.87	89.05
Jani et al (2019) [46]	MobileNetV2 [59]	88.53	88.51	88.34	88.69
Lee et al (2019) [39]	ResNet50 [56]	89.55	90.60	90.70	90.50
Vezakis et al (2019) [40]	ResNet18 [56]	89.95	90.35	90.72	89.99
Owais et al (2019) [10]	CNN^c^ + LSTM^d^ [28,56]	92.57	93.41	94.58	92.28
Cho et al (2019) [42]	InceptionResNet [60]	84.78	84.53	84.15	84.92
Dif et al (2020) [38]	ShuffleNet [61]	89.63	89.14	88.67	89.63
Song et al (2020) [43]	DenseNet201 [27]	92.12	92.42	92.91	91.93
Guimarães et al (2020) [37]	VGG16 [54]	85.72	85.80	86.24	85.37
Hussein et al (2020) [41]	ResNet101 [56]	90.24	91.14	91.52	90.78
Klang et al (2020) [47]	Xception [62]	86.05	84.88	84.19	85.58
Proposed method	DenseNet + LSTM + PCA^e^ + KNN^f^	96.19	96.99	98.18	95.86

^a^mAP: mean average precision.

^b^mAR: mean average recall.

^c^CNN: convolutional neural network.

^d^LSTM: long short-term memory.

^e^PCA: principal component analysis.

^f^KNN: k-nearest neighbor.

Furthermore, we iteratively analyzed and compared the sensitivity performance of our proposed method and all the baseline methods. For this purpose, an experimental setup known as a Monte Carlo simulation [63] was carried out, in which the testing performance of each model was evaluated by randomly selecting 20% of testing samples as an intermediate testing data set. The entire experiment was repeated a total of 200 times for both folds of the cross-validation (100 iterations for each fold), and we obtained 200 different performance results for each performance metric. We then calculated the average and standard deviation (for each metric), which presented the overall sensitivity performance of each method. Figure S11 in Multimedia Appendix 1 shows the comparative sensitivity results of our proposed method and the baseline methods. Based on these results, it can be concluded that the overall sensitivity performance of our proposed classification network was substantially higher than those of the existing networks.

We further performed a 2-tailed *t* test [64] and Cohen *d* [65] analysis to determine the significance of the performance gain of our proposed method compared with the second-best [10] and third-best [43] baseline methods. The sensitivity results (obtained in the previous section) were used to evaluate the performance of the *t* test and Cohen *d* analysis quantitatively. Generally, a *t* test analysis is carried out to magnify the performance difference of two models or algorithms in a quantitative way using a null hypothesis (*H*), which assumes that two systems are similar (ie, *H*=0). A rejection score (*P* value) between the two systems is calculated, which ultimately gives a confidence score for the rejection of this null hypothesis. In the Cohen *d* [65] analysis, the performance difference between two systems is determined by measuring effect size [66], which is normally categorized as small (approximately 0.2-0.3), medium (approximately 0.5), and large (≥0.8). A large effect size shows a significant performance difference between the systems. For our proposed method, we separately evaluated its rejection scores (*P* values) and effect sizes with the second-best and then the third-best baseline methods. The complete performance analysis results (for both the *t* test and Cohen *d*) are given in Table 3.

**Table 3:** The *t* test and Cohen *d* performance analysis results (*P* values and effect sizes).

Methods	Proposed vs second-best method				Proposed vs third-best method			
	Accuracy	F1	mAP^a^	mAR^b^	Accuracy	F1	mAP	mAR
*P* value	<.001	<.001	<.001	<.001	<.001	<.001	<.001	<.001
Cohen *d*	2.51	1.78	1.89	1.55	3.4	2.32	2.88	1.89

^a^mAP: mean average precision.

^b^mAR: mean average recall.

**Figure 5:**
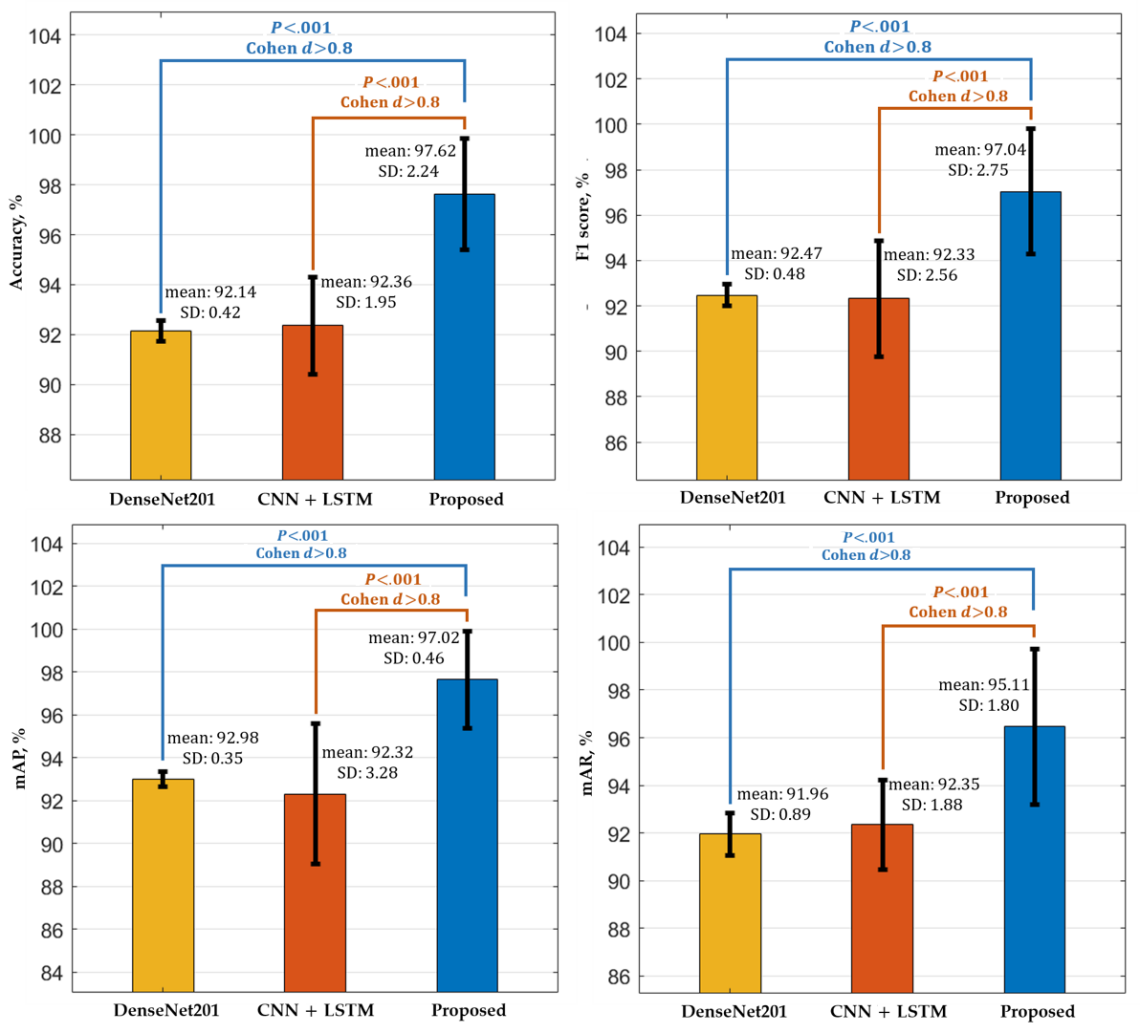
The *t* test and Cohen *d* performance comparison of our method with the second-best and third-best models with the average accuracy, F1 score, mAP, and mAR. CNN: convolutional neural network; LSTM: long short-term memory; mAP: mean average precision; mAR: mean average recall.

As seen in Table 3, the *P* values (*t* test analysis) are less than .001 for all performance metrics, which indicates that the null hypothesis is rejected (ie, *H*≠0), with a 99% confidence interval. Similarly, the effect sizes (Cohen *d* analysis) are higher than 0.8 for all the performance metrics. These 2 performance analysis results indicate that our proposed method shows a significant performance difference compared with both baseline methods. Moreover, Figure 5 presents the comparative performance (in mean, standard deviation, *P* value, and effect size) of our proposed method in comparison with the second-best and third-best models. The higher mean performance of our method (according to all the performance metrics) shows its superiority over the second-best and third-best baseline model.

Finally, we made a detailed comparison of our proposed network with various handcrafted feature–based methods. We evaluated the performance of our selected data set using 3 conventional handcrafted feature extraction methods—local binary pattern (LBP) [67], histogram of oriented gradients (HOG) [68], and multilevel local binary pattern (MLBP) [69]—with 4 different classifiers—AdaBoostM2 [32], Multi-SVM [33], random forest (RF) [70], and KNN [31]. In total, we obtained the classification performance of 12 different methods, as shown in Table 4. Among all these methods, the HOG feature extractor followed by the RF classifier showed the highest performance, with an average accuracy of 61.41%, F1 score of 63.19%, mAP of 68.66%, and mAR of 58.55%. This means the HOG features extractor method exploits more distinctive low-level features (ie, corners, blobs, or edges) than LBP or MLBP. In addition, the tree structure of the RF classifier further improved the classification decisions, which ultimately resulted in better classification performance. However, our proposed method performed substantially higher than the best handcrafted feature–based method (ie, HOG features extractor with RF classifier). In conclusion, our proposed network outperformed the various handcrafted and the deep features–based methods. We performed additional comparisons of KNN with more sophisticated classifiers, such as AdaBoostM2, Multi-SVM, and RF. In the case of our proposed network, KNN showed the best performance compared with AdaBoostM2, Multi-SVM, and RF, as shown in Table 4.

**Table 4:** Classification performance comparison of our proposed method with the other handcrafted feature–based methods.

Feature descriptor and classifier			Accuracy, %		F1, %		mAP^a^, %		mAR^b^, %
Local binary pattern [67]									
	AdaBoostM2	35.74		27.70		35.74		22.61	
	Multi-SVM^c^	43.84		42.35		42.99		41.72	
	RF^d^	57.10		53.85		54.79		52.95	
	KNN^e^	50.46		47.36		46.86		47.87	
Histogram of oriented gradients [68]									
	AdaBoostM2	39.35		32.86		39.35		28.22	
	Multi-SVM	49.84		53.80		67.39		44.88	
	RF	61.41		63.19		68.66		58.55	
	KNN	53.20		54.68		58.41		51.45	
Multilevel local binary pattern [69]									
	AdaBoostM2	44.02		37.45		44.02		32.59	
	Multi-SVM	55.47		53.10		54.75		51.55	
	RF	61.40		57.57		59.08		56.13	
	KNN	55.40		52.20		52.06		52.33	
Proposed feature descriptor (DenseNet + LSTM^f^ +PCA^g^)									
	AdaBoostM2	93.39		93.66		94.35		92.98	
	Multi-SVM	95.50		96.43		97.98		94.96	
	RF	81.16		82.96		84.48		81.55	
	KNN	96.19		96.99		98.18		95.86	

^a^mAP: mean average precision.

^b^mAR: mean average recall.

^c^SVM: support vector machine.

^d^RF: random forest.

^e^KNN: k-nearest neighbor.

^f^LSTM: long short-term memory.

^g^PCA: principal component analysis.

## Discussion

### Principal Findings

**Figure 6:**
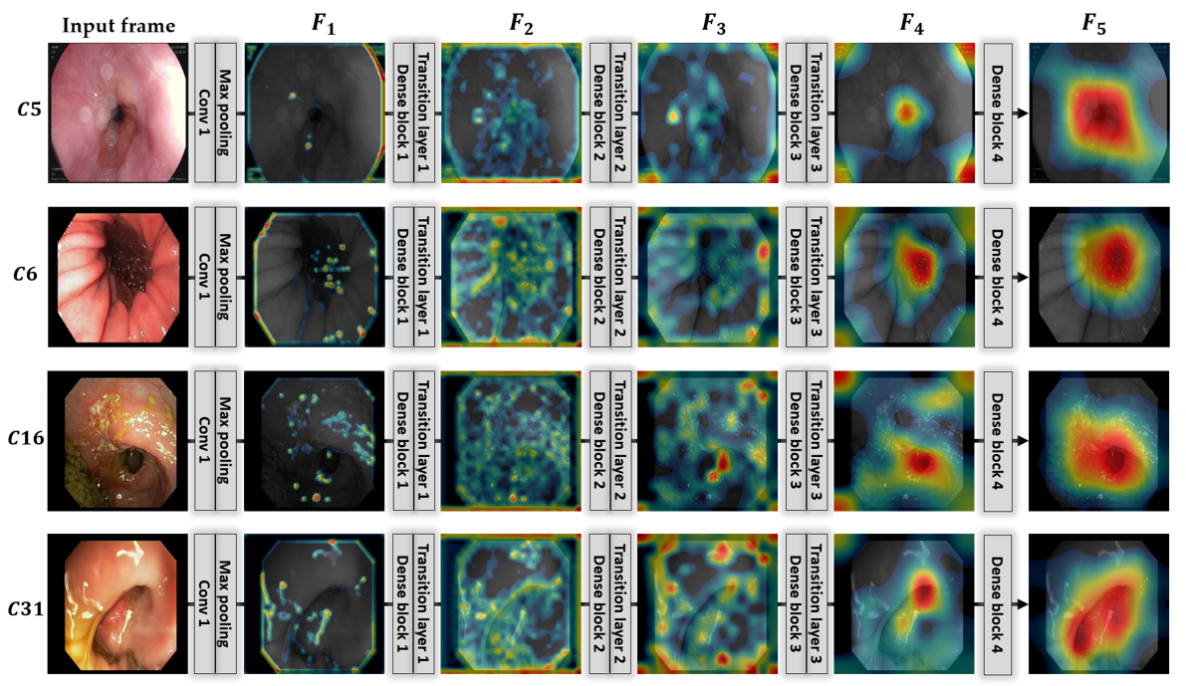
Obtained class-specific discriminative regions from different parts of the first-stage network (DenseNet) for given input frames.

In this research, we used the strength of recent ANNs in endoscopy and proposed a high-performance CAD framework to diagnose multiple GI diseases simultaneously in a given endoscopic video. First, we implemented an efficient video classification network to classify endoscopic videos into one of 37 different categories (including both normal and diseased classes). Our proposed network contemplates both spatial and temporal features, which ultimately resulted in better performance in comparison with other modern classification networks. The spatial features are extracted in the first step using a densely connected convolutional network, and then an LSTM-based network further processes the spatial features to extract the temporal information. Therefore, optimal spatial features must be extracted to achieve the best performance by the LSTM-based network in the second stage. For this purpose, we considered a DenseNet model, which shows superior classification performance compared with various CNN models. The performance difference between DenseNet and other CNN models can be observed in Table 2. These results show that our selected DenseNet model had a performance gain of 1.88% in average accuracy compared with the second-best CNN model (ResNet101). It exploits the optimal spatial features by processing the input frames through different dense blocks and transition layers, highlighting the class-specific discriminative regions [71] as the optimal feature maps. The presence of dense blocks and transitions layers in DenseNet makes it different from the other CNN models and results in superior performance. Figure 6 presents the progress of class-specific discriminative regions (activation maps) through the different parts of the first-stage network. For better interpretation, we calculated an average activation map for each layer and represented it with a pseudocolor scheme (red as the maximum value and blue as the minimum value) by overlaying it on the input frame. As shown in Figure 6, the class-specific discriminative regions (activation maps F1, F2, …, F5) become more prominent after passing through the different layers of the network of Figure 2. Ultimately, we can obtain class-specific regions (activation map F5) that include the particular visual pattern for each class. These final class-specific activation maps are processed by the LSTM-based network for temporal feature extraction after passing through the global average pooling layer of the first-stage network. The key difference between DenseNet [27] and the LSTM-based network [10,28] is the extraction of 2 different types of features from the given input video sequence. The LSTM-based network exploits the time-dependent features of the successive frames of a video, while DenseNet only extracts the features within a single frame. Because of these 2 different types of features, the networks are different from each other. However, their combined connectivity generates an optimal representation of the given input video of *n* consecutive frames (ie, *I*_1_, *I*_2_, *I*_3_ …, *I_n_*) in terms of spatiotemporal features, which ultimately results in better classification performance. The significant performance gain by our proposed network boosts its usability in the diagnosis of various GI diseases by automatically detecting and classifying various types of GI diseases, such as gastric ulcers, cancer, or polyps. In the second step, these classification results were further used to retrieve relevant cases (endoscopic frames) from the database that are closely related to the current medical condition of a patient. Our proposed method is based on class prediction–based retrieval, in which feature matching is performed only within the predicted class to find the best matches. However, without class prediction–based retrieval, feature matching is done with the entire database, which is time-consuming.

We computed the retrieval performance for both methods (with and without class prediction) using the proposed and second-best baseline model [10], as presented in Table 5. These comparative results showed a substantial performance difference between our proposed method and the second-best method for both cases (with and without class prediction). The retrieval performance of our proposed class prediction–based retrieval method was also better than the performance of the method without class prediction–based retrieval. In addition to the performance difference, the main advantage of our class prediction–based retrieval method is the optimal features comparison time required to retrieve the relevant cases from the database. In the method without class prediction, this feature comparison time was significantly higher because of the large number of feature comparisons, as the number of feature comparisons is directly related to the volume of the whole data set (ie, the total number of available data samples in the data set). Moreover, this feature comparison time also increases with the increasing number of data samples in each class. On the other hand, in our proposed class prediction–based retrieval method, there is no relation between the feature comparison time and the volume of the data set. Hence, our proposed retrieval method is more efficient in terms of retrieval computational cost.

**Table 5:** Performance comparisons of our proposed and the second-best baseline method [10] using both retrieval methods.

Method	With class prediction					Without class prediction			
	Accuracy, %	F1, %	mAP^a^, %	mAR^b^, %	Accuracy, %		F1, %	mAP, %	mAR, %
Owais et al [10]	92.57	93.41	94.58	92.28	93.18		94.02	94.68	93.38
Proposed	96.19	96.99	98.18	95.86	96.13		96.94	98.04	95.89

^a^mAP: mean average precision.

^b^mAR: mean average recall.

**Figure 7:**
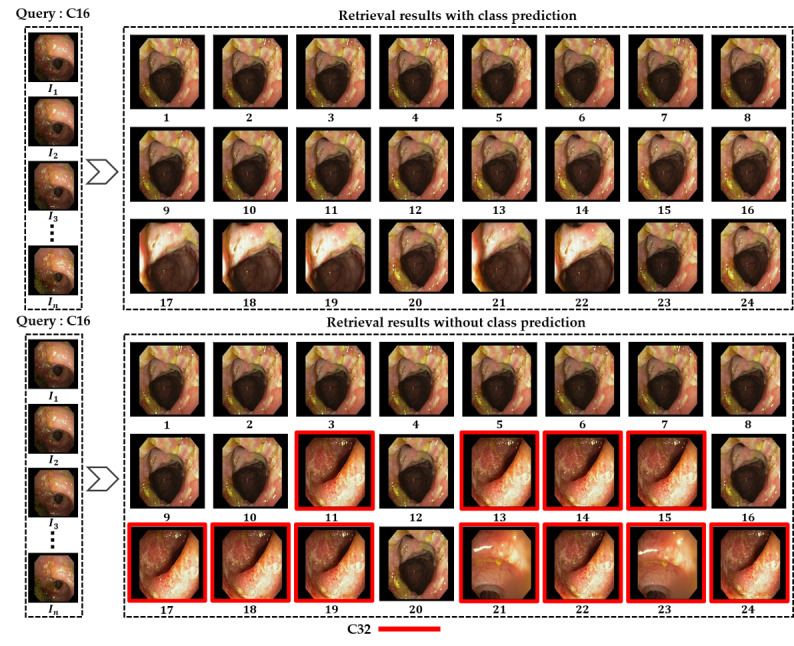
Obtained retrieval results in ranked order (1st to 24th best matches) for the C16 input query frame using both a class prediction–based method and a method without class prediction.

**Figure 8:**
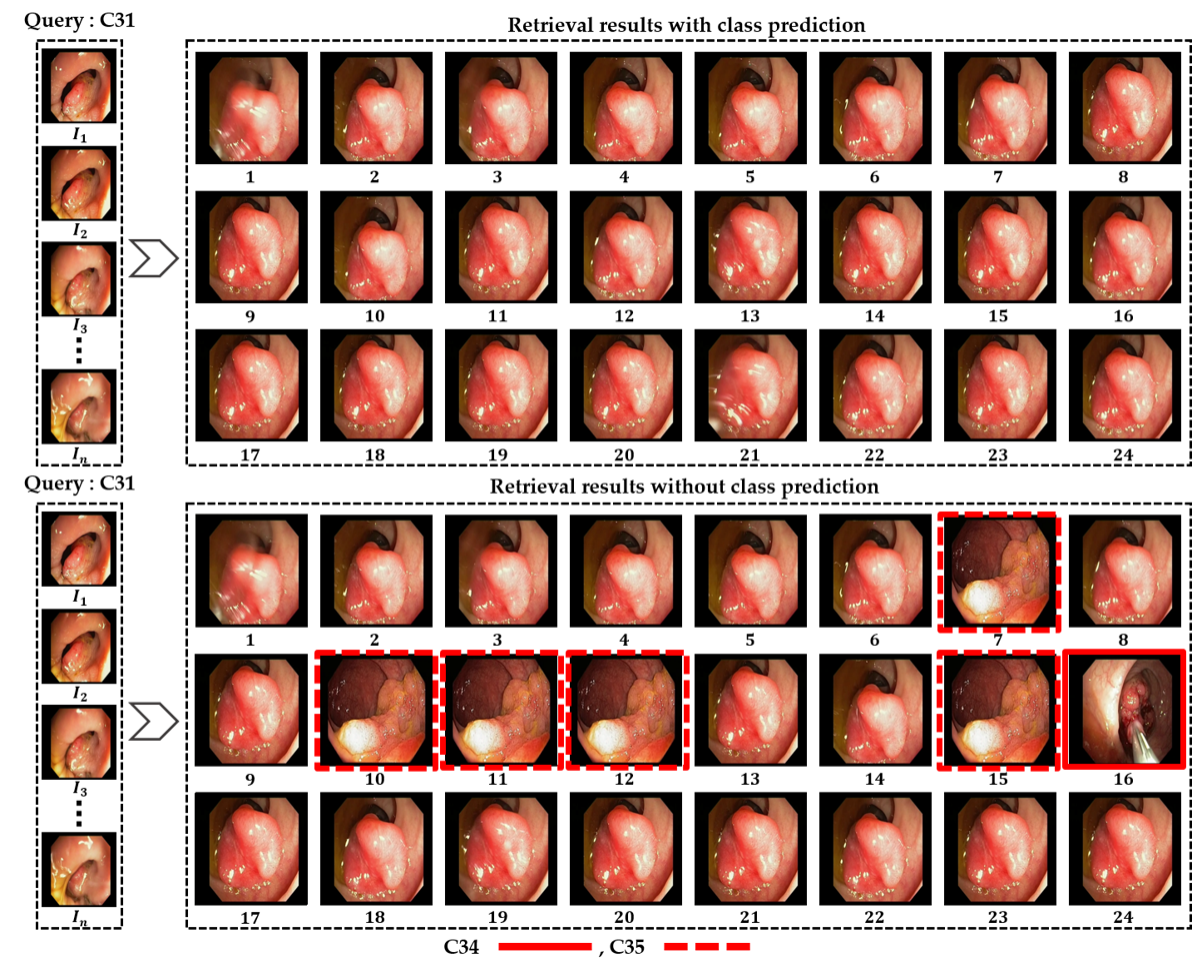
Obtained retrieval results in ranked order (1st to 24th best matches) for the C31 input query frame using both a class prediction–based method and a method without class prediction.

In addition, Figures 7 and 8 show the retrieved frames for the given input query from classes C16 and C31, respectively. All these results were computed separately for both retrieval methods (our proposed class prediction–based method and other methods without class prediction) to show the performance difference visually. All the results are presented in ranked order by retrieving the 24 best matches for each input query. As seen in the figures, our proposed method retrieves all the best matches as true positives for the given input query, whereas the methods without class prediction show many false-positive frames (highlighted with a red bounding box) among the retrieved frames. Hence, the class prediction–based retrieval method outperforms the other method in retrieving multiple best matches, and the retrieval methods without class prediction show performance degradation in retrieving multiple best matches.

### Limitations and Future Work

Due to a limited data set, data splitting was performed by considering the first half of the consecutive frames of a video as the training data and the remaining half as the testing data. Thus, the testing data set was obtained from the same sources as the training data set, which may raise issues of the generalizability of our framework. However, we accomplished our goal to incorporate a large number of GI diseases in a single deep learning–based CAD framework and provided an initial pretrained network in the field of GI diagnostics. To highlight the superiority of our proposed solution, we used a similar data splitting and experimental protocol to evaluate the results of various existing methods [5,8,10,36-47]. Finally, we provided a novel baseline solution in the emergent clinical setting as a supporting tool that can be further evolved in future studies.

According to our experimental analysis, a database of sufficient size with all of the common diseases can further enhance the generalizability of our proposed framework. In future work, we will further explore a large number of cases with this data set and try to further enhance the overall performance of the system by using different videos in training and testing.

### Conclusions

This study presented a comprehensive CAD tool, a deep learning–based classification-driven retrieval framework, for identifying various types of GI diseases. The complete framework comprises a deep learning–based classification network followed by a retrieval method. The classification network predicts the disease type for the current medical condition, and the retrieval part then shows the relevant cases from the previous database. As a result, past cases help the medical expert subjectively validate the current prediction by the CAD method, which ultimately results in better diagnosis and treatment. In the case of a wrong prediction by the computer, the medical expert can check other relevant cases (ie, second-, third-, or fourth-best matches), which may be more relevant than the first-best match. Our results (also provided in Multimedia Appendix 2) show the superiority of our proposed method over various other state-of-the-art methods.

## Appendix

Multimedia Appendix 1 Other supplementary material.

Multimedia Appendix 2 Experimental results.

## Abbreviations

ANN: artificial neural network
CAD: computer-aided diagnosis
CNN: convolutional neural network
GI: gastrointestinal
HOG: histogram of oriented gradients
KNN: k-nearest neighbor
LBP: local binary pattern
LSTM: long short-term memory
mAP: mean average precision
mAR: mean average recall
MLBP: multilevel local binary pattern
PCA: principal component analysis
RF: random forest
SVM: support vector machine
