# Supplementary material for: Automated Diagnosis of Various Gastrointestinal Lesions Using a Deep Learning–Based Classification and Retrieval Framework With a Large Endoscopic Database: Model Development and Validation
Source: J Med Internet Res. 2020 Nov 26;22(11):e18563. doi: 10.2196/18563 (PMC7728528; doi:10.2196/18563)

**Multimedia Appendix 1:** Other supplementary material

# **Automated Diagnosis of Various Gastrointestinal Lesions Using a Deep Learning–Based Classification and Retrieval Framework With a Large Endoscopic Database: Model Development and Validation**

**Authors**

Muhammad Owais, MSc; Muhammad Arsalan, PhD; Tahir Mahmood, MSc; Jin Kyu Kang, BSc; Kang Ryoung Park, PhD

**Affiliations:**

Division of Electronics and Electrical Engineering, Dongguk University, Seoul, Korea

Emails: malikowais266@gmail.com (M.O); arsal@dongguk.edu (M. A.); tahirmahmood.cs@gmail.com (T.M.); kangjinkyu@dgu.edu (J. K. K.)

**Corresponding Author:**

Kang Ryoung Park, PhD

Division of Electronics and Electrical Engineering

Dongguk University

30 Pildong-ro 1-gil, Jung-gu

Seoul, 04620

Republic of Korea

Phone: 82 10 3111 7022

Fax: 82 2 2277 8735

Email: parkgr@dgu.edu

## Related Work

Over the past few years, a considerable contribution was made by robust and efficient CAD tools for the endoscopy domain. However, most of these methods are designed to detect specific types of GI diseases such as polyps, ulcers, tumors, or cancer by using hand-crafted or deep features-based approaches. Before the advent of deep features-based methods, most studies used hand-crafted features such as color and texture information to perform the automated detection and classification of particular types of GI disease [18-24]. For example, Barbosa et al. [18] proposed a texture feature-based tumor detection method within the small intestine. A wavelet transform was employed to extract the most significant texture information, which was further used by a multilevel perceptron (MLP) network to perform the final tumor detection task. Karargyris et al. [19] presented a synergistic methodology for detecting abnormal patterns such as polyps and ulcers in the small intestine. A multilevel feature extraction method was proposed by considering color, texture, and geometric features to characterize such types of abnormalities. The final detection was performed based on these multilevel features by using a fuzzy support vector machine (SVM).

Another texture feature-based polyp detection approach was presented in [20] by utilizing the advantages of wavelet and uniform local binary pattern (ULBP) for optimal feature extraction. Furthermore, these extracted features were fed to the SVM to categorize normal and abnormal tissues. The extracted features show robustness to the illumination changes and provide the scale-invariant capability, which is the main advantage of this proposed method. Based on this work, Le et al. [21] introduced another color texture feature-based approach to perform tumor recognition in wireless capsule endoscopy (WCE) images. A similar integrated approach using ULBP and wavelets was adopted to characterize WCE images, and then an SVM was trained to distinguish tumor and nontumor images. This method also showed robustness to variations in scale and illumination due to the multiscale wavelet analysis. Furthermore, Manivannan et al. [22] proposed a general classification framework related to colonoscopy to categorize each video frame either as a normal or an abnormal image. In this proposed method, a scale-space method was adopted to extract multi-resolution texture features that showed robustness to noise and illumination changes. To do this, a multi-resolution local binary pattern algorithm was applied to a set of multi-resolution images. Later, another variant of a local binary pattern (LBP) feature descriptor called the extended Gaussian filter local binary pattern (xGF-LBP) was proposed to classify similar colonoscopy video frames either as normal or abnormal images [23]. This newly proposed feature descriptor was also robust to noise and changes in illumination and captured more informative edge-like features for better classification performance. Moreover, Yuan et al. [24] proposed a unified polyps detection method by improving the classification capability of the scale-invariant feature transform (SIFT) method. In this approach, various combinations of different texture feature extraction methods such as LBP, ULBP, complete local binary pattern (CLBP), and histogram of oriented gradients (HoG) were considered with SIFT features. All these combinations were classified by Fisher’s linear discriminant analysis (FLDA) and SVM classifiers. Finally, based on the obtained classification accuracies, it was concluded that the concatenation of CLBP and SIFT features with the SVM classifier resulted in better classification accuracy than all the other combinations.

In the recent few years, various deep learning-based CAD tools have been proposed in the endoscopic video and image analysis domain. Such deep learning-based CAD tools are capable of performing the classification and detection of different GI abnormalities in a more precise and accurate way than the previous handcrafted features-based methods. For example, Seguí et al. proposed a well-trained deep CNN model for small intestine motility characterization [2]. The proposed CNN-based CAD system learned the generic characteristics of six different types of motility events from the vast collection of WCE videos. The final classification accuracies show the superiority of this method over various handcrafted features-based methods. In [3], another CNN-based CAD system was developed to analyze the presence of celiac disease quantitatively by using WCE videos. A well-known CNN model named as GoogLeNet was used to differentiate normal and abnormal (i.e., celiac disease) cases. The significant performance results illustrate its usefulness for better diagnosis of celiac disease patients. As deep learning is a data-driven approach that requires a sufficient amount of data to train a network, a transfer learning approach was applied by Zhang et al. [4] to develop an efficient deep learning-based framework for colorectal polyp detection and classification. In this approach, two different CNN models were initially trained using general classification and detection dataset, and then further fine-tuning was performed for the colorectal polyp dataset. The first CNN model was trained to detect the polyp image, and then the second CNN model further categorized the polyp image either as an adenomatous colorectal polyp or a hyperplastic polyp type. The experimental results showed the superiority of the proposed CNN-based diagnostic tool. However, because of the high computational cost of the system, the polyp detection task could not be performed in a real-time environment. Later, another real-time colorectal polyp classification framework was proposed by Byrne et al. [5] based on a single deep CNN model. A pre-trained CNN model was used to categorize each input frame as one of four different types, labeled adenomatous polyp, hyperplastic polyp, no polyp, or unsuitable. The experimental results demonstrate the computational efficiency of this classification framework in a real-time environment.

In a study by Shichijo et al. [6], a CNN-based diagnostic tool was proposed for the detection of H. pylori infection in the GI tract. In this study, two different CNN models were used to predict two different class labels for the given endoscopic image. The first CNN model detected H.pylori infection by classifying the image either as a positive (infected) or negative (not infected) sample. Then, the second CNN model further assigned an anatomical location to the image by classifying it into one of eight different anatomical location classes. In this way, classification-based detection and localization were performed to diagnose H. pylori infection more effectively. In [7], the authors proposed an automatic polyp detection framework by using three-dimensional (3D) deep CNN models. In this framework, two 3D-CNNs models were connected in a cascading manner to reduce the number of false positives and enhance polyp detection performance. The polyp detection performance shows the superiority of this 3D CNN-based framework in comparison with a simple two-dimensional CNN and various hand-crafted feature-based methods. In a recent study conducted by Takiyama et al. [8], a pre-trained CNN architecture named GoogLeNet was used to classify the given endoscopic images into different anatomical locations. In this classification task, there were four key anatomical locations, labeled as larynx, esophagus, stomach, and duodenum. Then, the stomach class was further divided into three sub-categories labeled as upper, middle, and lower regions. In another study conducted by Hirasawa et al. [9], a single shot multibox detector (SSD) method was used to detect gastric cancer in endoscopic images. The detection capability of the proposed method was substantially higher than conventional hand-crafted feature-based methods. However, it required high-quality endoscopic images to achieve accurate cancer detection.

Because the internal structure of the human GI tract is captured as a moving sequence (video) with respect to time during an endoscopy procedure. Therefore, an endoscopic video encompasses both spatial and temporal information. In a video, the temporal information exists among the sequence of consecutive frames and provides essential information. Therefore, it is possible to use both spatial and temporal information in developing a high-performance CAD tool with good diagnostic capability. However, previous deep learning-based CAD methods used only the spatial information for the automatic diagnosis of GI diseases, which reduced the overall diagnostic performance. In addition, a limited contribution was made in developing a comprehensive CAD tool to detect multiple GI diseases in the whole GI tract. In our previous study, we investigated a first multiple GI disease classification framework using both spatial and temporal information [10]. The main objective of this method was to classify the given endoscopic video frames into one of the 37 different categories, which included both diseased and normal human GI tract cases. However, the classification and retrieval performance of this previous method was limited. In our current study, we proposed another spatiotemporal feature-based classification and retrieval framework to further enhance the performance of the previously proposed method [10]. Our proposed method reduced the feature dimension up to 95% (i.e., from 1×600 to 1×31) in comparison with our previous work [10] with the performance gain of 3.62% in terms of average accuracy. Such a significant performance gain of our newly proposed method makes it distinctive from our previous work [10]. In addition, our research used DensNet+LSTM with PCA and KNN different from previous method [10] using ResNet+LSTM without PCA and KNN, and in our proposed framework, the number of successive frames to be classified can be variable (i.e., n=1,2,3,…,150) rather than using the fixed-length sequence of previous method [10].

In this framework, the classification part is used to detect the abnormal conditions in endoscopy video and the retrieval part shows the past cases related to the current condition. In this way, past cases can help the medical expert to validate the current prediction by the CAD method in a subjective way, which ultimately result is better diagnosis and treatment. The strengths and weaknesses of our proposed and various state-of-the-art studies are summarized in Table S1 in Multimedia Appendix 1.

**Table S1*.*** A comparison summary of our proposed and existing baseline methods developed for the classification and detection of the various types of GI diseases.

| **Method** | | **Purpose** | **No. of Classes** | **Strength** | **Weakness** |
| --- | --- | --- | --- | --- | --- |
| Hand-Crafted Features | Wavelet-based texture features + MLP [18] | Tumor detection in the small bowel | 2 | High sensibility and specificity | Limited dataset and number of classes |
|  | Color, texture, and geometric features + SVM [19] | Polyp and ulcer detection in the small bowel | 2 | High sensitivity and computationally efficient | Low specificity and detection performance  Limited dataset and number of classes |
|  | ULBP and wavelet-based texture features + SVM [20] | GI polyp detection | 2 | Scale-invariant and robust to variations in illumination | Low performance as well as limited dataset and number of classes |
|  | ULBP and wavelet-based texture features + SVM [21] | GI tumor recognition | 2 | Scale and illumination invariant | Overall low performance as well as limited dataset and number of classes |
|  | mLBP-based multi-scale texture features + SVM [22] | Normal and abnormal frame classification for colonoscopy | 2 | High classification performance  Robust to noise and variations in illumination | Perform general categorization (i.e., normal and abnormal images) |
|  | xGF-LBP based multi-scale texture features + SVM [23] | Normal and abnormal frame classification for colonoscopy | 2 | High classification performance  Robust to noise and variations in illumination | Perform general limit categorization (i.e., normal and abnormal images) |
|  | Multiple texture features (SIFT, HoG, LBP, CLBP, ULBP) + FLDA, SVM [24] | GI Polyp detection | 2 | High classification performance  Scale-invariant | Limited dataset and number of classes |
| Deep Features | CNN [2] | Classification of different small intestine motilities | 6 | High classification performance | Classify a limited number of categories related to a specific part of GI tract |
|  | CNN [3] | Classification of celiac disease | 2 | High specificity and sensitivity | Limited number of classes and dataset |
|  | CNN [4] | Detection and classification of colorectal polyps | 3 | High detection accuracy | Limited number of classes and dataset  Low classification accuracy |
|  | CNN [5] | Real-time analysis of colorectal polyp type | 4 | High sensitivity and accuracy | Limited number of classes  Low specificity |
|  | CNN [6] | Detection of H. pylori infection | 9 | Comparable accuracy of second CNN with the clinical diagnosis reference standard | Accuracy of CAD should be enhanced  Limited number of classes |
|  | Online and offline 3D-CNN [7] | Colorectal polyp detection | 2 | Computationally efficient | CAD performance should be enhanced |
|  | CNN [8] | Anatomical classification of GI images | 6 | High classification accuracy  Low processing time | Limited number of classes  Used only for anatomical classification |
|  | CNN-based SSD detector [9] | Gastric cancer detection | 2 | High sensitivity  Low processing time | Required high-quality images  Limited dataset and number of classes |
|  | CNN (ResNet) + LSTM [10] | Classification of multiple GI diseases | 37 | Low processing time  High classification accuracy | Sequential training of CNN and LSTM requires more time |
|  | DenseNet + LSTM + PCA + KNN  (Proposed) | Classification and retrieval of multiple GI diseases | 37 | Best classification accuracy  Significant low features dimension | Lack of end-to-end training |

**Table S2.** Layer-wise configuration details of DenseNet and LSTM network.

| **DenseNet (growth rate (k) = 32)** | | | | |
| --- | --- | --- | --- | --- |
| **Layers Name** | **Output Feature Map Size** | **Kernel Size** | **Number of Iterations (*l*)** | **Learnable Parameters** |
| Image Input | $224\times224 \times3$ | - | - | - |
| Conv1 | $112\times112\times64$ | $7\times7 conv$ | 1 | 9,600 |
| Max Pooling | $56\times56\times64$ | $3\times3\max pool$ | 1 | - |
| Dense Block 1 | $56\times56\times256$ | $\begin{matrix} 1\times1 conv \\ 3\times3 conv \end{matrix}$ | 6 | 336,000 |
| Transition Layer 1 | $28\times28\times128$ | $\begin{matrix} 1\times1 conv \\ 2\times2 avg pool \end{matrix}$ | 1 | 33,408 |
| Dense Block 2 | $28\times28\times512$ | $\begin{matrix} 1\times1 conv \\ 3\times3 conv \end{matrix}$ | 12 | 921,600 |
| Transition Layer 2 | $14\times14\times256$ | $\begin{matrix} 1\times1 conv \\ 2\times2 avg pool \end{matrix}$ | 1 | 132,352 |
| Dense Block 3 | $14\times14\times1792$ | $\begin{matrix} 1\times1 conv \\ 3\times3 conv \end{matrix}$ | 48 | 8,079,360 |
| Transition Layer 3 | $7\times7\times896$ | $\begin{matrix} 1\times1 conv \\ 2\times2 avg pool \end{matrix}$ | 1 | 1,610,112 |
| Dense Block 4 | $7\times7\times1920$ | $\begin{matrix} 1\times1 conv \\ 3\times3 conv \end{matrix}$ | 32 | 6,983,680 |
| Avg Pooling | $1\times1\times1920$ | $7\times7 avg pool$ | 1 | - |
| Fully Connected | $1\times1 \times37$ | - | - | 1,924,840 |
| Softmax | $1\times1 \times37$ | - | - | - |
| Classification Output | 37 | - | - | - |
| **LSTM Network** | | | | |
| Sequence Input | $n\times1\times1 \times1920$ | - | - | - |
| LSTM | $1\times1 \times600$ | - | - | 1,951,200 |
| Dropout | $1\times1 \times600$ | - | - | - |
| Fully Connected | $1\times1 \times37$ | - | - | 22,237 |
| Softmax | $1\times1 \times37$ | - | - | - |
| Classification Output | $37$ | - | - | - |
| **Total number of parameters: 22,004,389** | | | | |

**Table S3.** Details of multiple sub-categories of each anatomical area with the actual class description.

| **Gastrointestinal Tract** | | **Class Name**  **(Normal/Disease Cases)** | **Number of Patients** | **Total Number of Frames** |
| --- | --- | --- | --- | --- |
| **Anatomical District** | **Sub-category** |  |  |  |
| Esophagus | Larynx | C1: Normal | 2 | 774 |
|  | Upper part | C2: Normal  C3: Esophageal candidiasis  C4: Esophageal papillomatosis | 1  1  1 | 1250  838  544 |
|  | Lower part (z-line) | C5: Normal | 1 | 500 |
| Stomach | Cardia | C6: Hiatal hernia | 1 | 1296 |
|  | Fundus | C7: Atrophic gastritis  C8: Atrophic and xanthoma gastritis | 1  1 | 482  509 |
|  | Body | C9: Benign hyperplastic polyps  C10: Adenocarcinoma (Cancer) | 3  2 | 2140  1910 |
|  | Pylorus | C11: Normal | 2 | 2550 |
| Small Intestine | Duodenum | C12: Normal  C13: Ulcer  C14: Papilla Vateri | 1  5  2 | 846  2690  1404 |
|  | Terminal Ileum | C15: Crohn’s disease | 1 | 1680 |
|  | Ileocecal | C16: Severe Crohn’s disease | 1 | 556 |
|  | Ileocecal valve | C17: Crohn’s disease | 2 | 1676 |
| Large Intestine | Caecum | C18: Adenocarcinoma (Cancer)  C19: Melanosis coli  C20: Cecal angioectasia  C21: Appendix aperture | 3  1  2  2 | 2602  684  807  1388 |
|  | Ascending/  Transverse/Descending Colon | C22: Adenocarcinoma (Cancer)  C23: Melanosis coli  C24: Different types of polyps  C25: Dyed resection margins  C26: Dyed lifted polyps  C27: Melanosis coli and tuber adenoma  C28: Inflammatory polyposis  C29: Normal | 8  2  1  1  1  1  1  1 | 2586  1207  500  500  500  486  764  1000 |
|  | Sigmoid Colon | C30: Tuber adenoma  C31: Polypoid cancer | 6  1 | 4424  564 |
|  | Rectosigmoid | C32: Ulcerative colitis | 6 | 4142 |
| Rectum | - | C33: Severe Crohn’s disease  C34: Adenocarcinoma (Cancer)  C35: Tuber adenoma  C36: Normal  C37: A focal radiation injury | 1  7  2  1  1 | 2148  2724  2138  840  822 |

**Table S4.** Selected global training parameters in our experiments for the training of both networks.

| **Networks** | **Number of**  **Epochs** | **Mini-Batch Size** | **Learning Rate** | **Momentum Term** | **L2-Regularization** | **Learning Rate Drop Factor** |
| --- | --- | --- | --- | --- | --- | --- |
| DenseNet | 4 | 10 | 0.001 | 0.9 | 0.0001 | 0.1 |
| LSTM | 10 | 50 | 0.0001 | 0.9 | 0.0001 | 0.1 |

**Table S5.** Structural and parametric comparisons between our proposed model and the other state-of-the-art ANN models.

| **CNN Models** | **Size (MB)** | **Network Depth** | **Parameters (Millions)** | **No. of Conv. Layers** | **No. of FC Layers** | **No. of LSTM Layers** | **Image Input Size** |
| --- | --- | --- | --- | --- | --- | --- | --- |
| SqueezeNet [57] | 4.6 | 18 | 1.24 | 18 | - | - | 227-by-227 |
| AlexNet [16] | 227 | 8 | 61 | 5 | 3 | - | 227-by-227 |
| GoogLeNet [3,8,58] | 27 | 22 | 7 | 21 | 1 | - | 224-by-224 |
| VGG19 [54] | 535 | 19 | 144 | 16 | 3 | - | 224-by-224 |
| VGG16 [54] | 515 | 16 | 138 | 13 | 3 | - | 224-by-224 |
| InceptionV3 [5,55] | 89 | 48 | 23.9 | 47 | 1 | - | 299-by-299 |
| MobileNetV2 [59] | 13 | 53 | 3.5 |  | - | - | 224-by-224 |
| ResNet50 [56] | 96 | 50 | 25.6 | 49 | 1 | - | 224-by-224 |
| ShuffleNet [61] | 6.3 | 50 | 1.4 |  | - | - | 224-by-224 |
| ResNet18 [56] | 44 | 18 | 11.7 | 17 | 1 | - | 224-by-224 |
| InceptionResNet [60] | 209 | 164 | 55.9 | 163 | 1 |  | 299-by-299 |
| ResNet101 [56] | 167 | 101 | 44.6 | 100 | 1 | - | 224-by-224 |
| Xception [62] | 85 | 71 | 22.9 | 70 | 1 | - | 299-by-299 |
| DenseNet201[27] | 77 | 201 | 20 | 200 | 1 | - | 224-by-224 |
| CNN+LSTM [10] | 47 | 19 | 13.17 | 17 | 1 | 1 | 224-by-224 |
| Proposed | 86 | 202 | 22 | 200 | 1 | 1 | 224-by-224 |

**Figure S1.** A simple workflow diagram of a computer-aided diagnosis tool to visualize its clinical usability in making an effective diagnostic decision. CAD: computer-aided diagnosis.


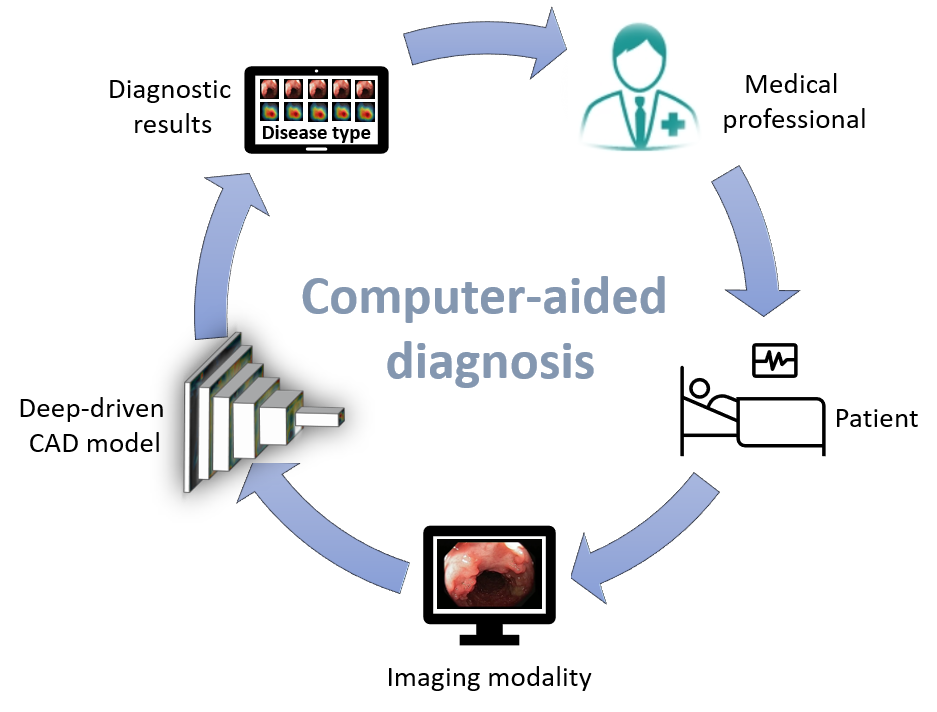


**Figure S2.** The complete structure of a single dense block followed by the transition layer. Note that each “Conv” layer is also followed by a batch normalization and rectified linear unit layer.


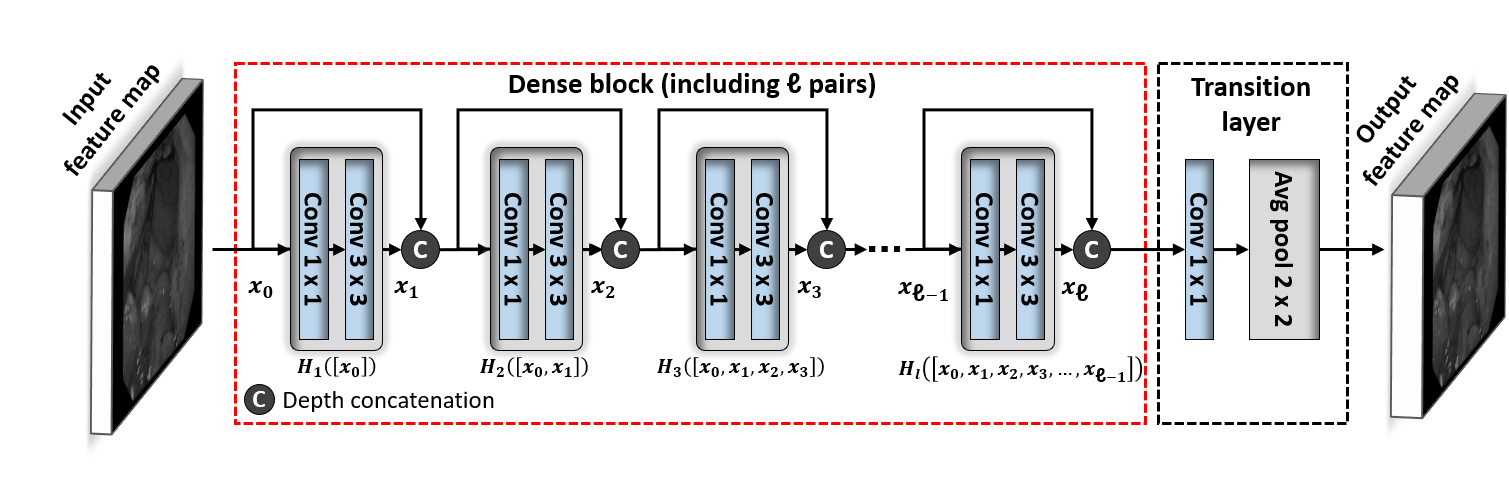


**Figure S3.** The conceptual visualization of all the intermediate data sets created by our proposed classification framework in different stages. KNN: k-nearest neighbor; LSTM: long short-term memory; PCA: principal component analysis.


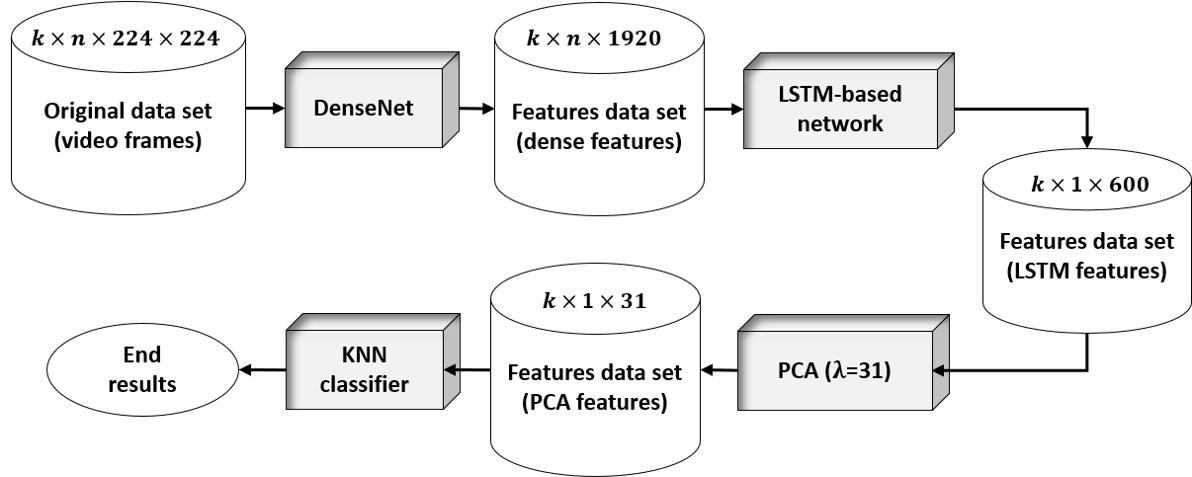


**Figure S4.** Example frames from our selected data set to visualize high intraclass variance.


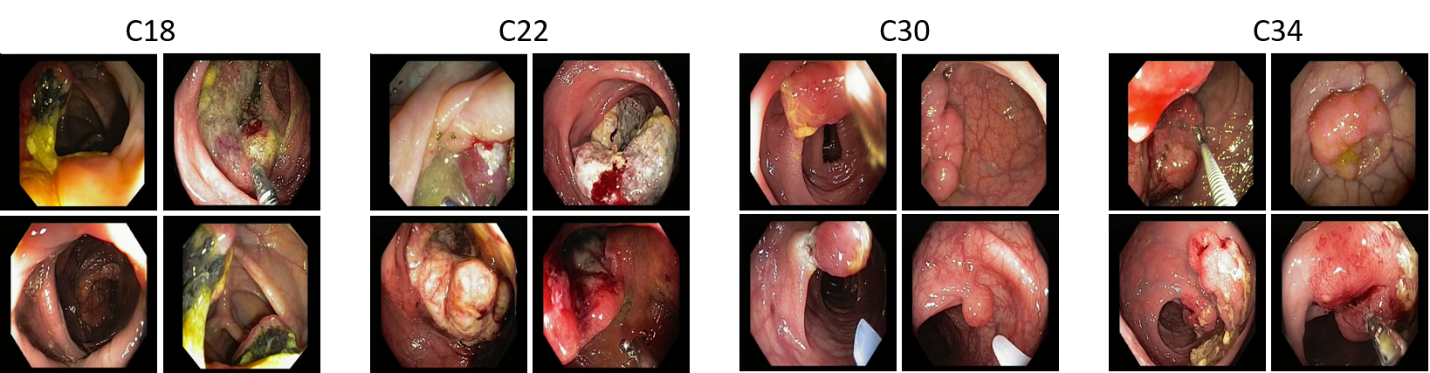


**Figure S5.** Example frames to visualize the significant difference between training and testing data for some classes that mainly consist of single-patient data (ie, one video per class).


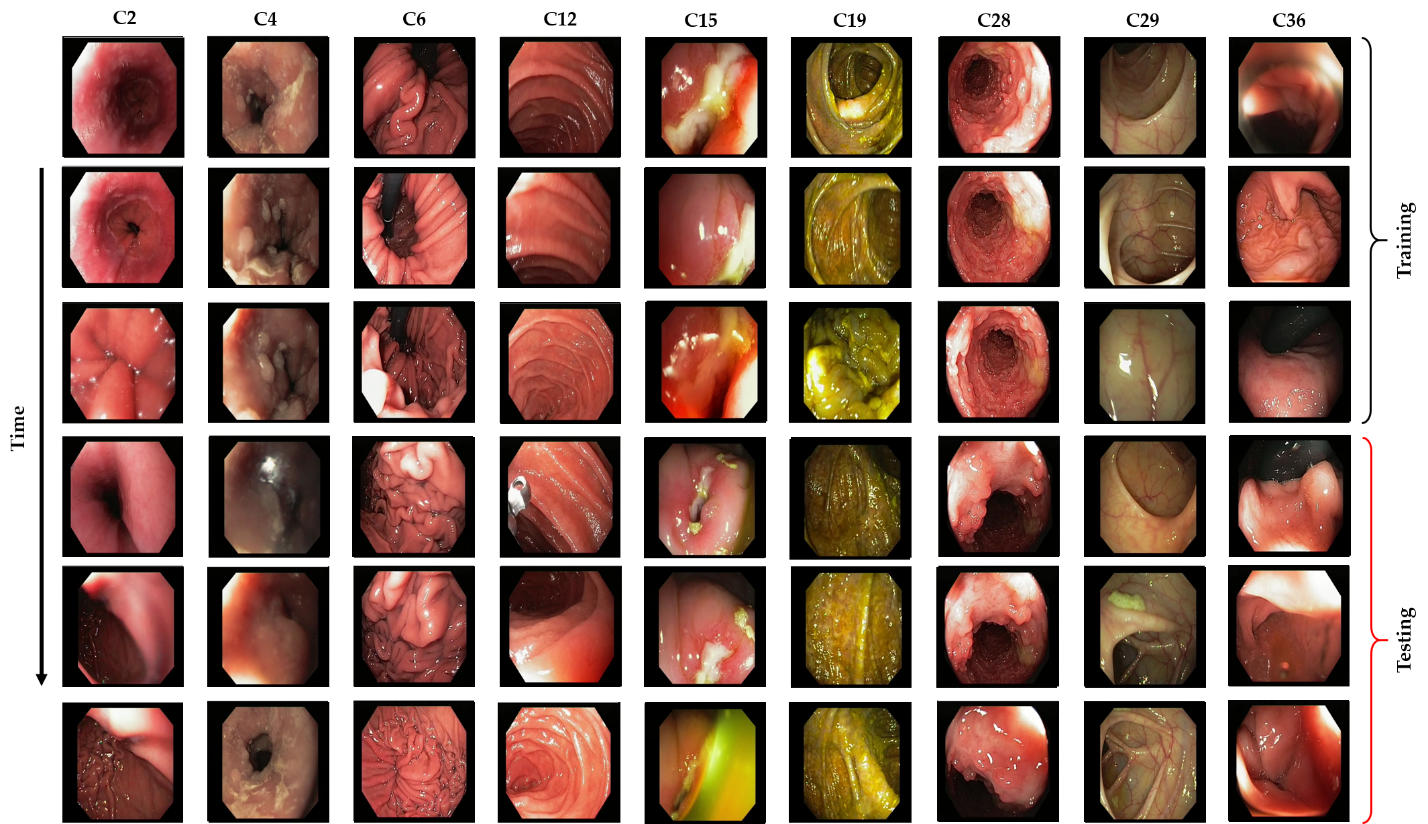


**Figure S6.** Progress of training accuracy and loss according to the number of epochs during the first stage (ie, training of the DenseNet for spatial features extraction).


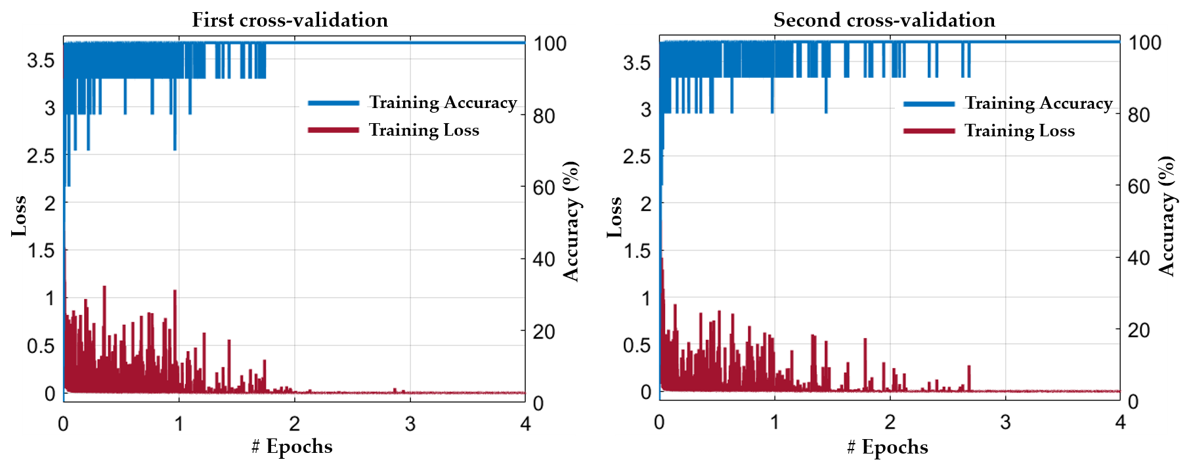


**Figure S7.** Progress of training accuracy and loss according to the number of epochs during the second stage (i.e., training of LSTM-based network for temporal features extraction).


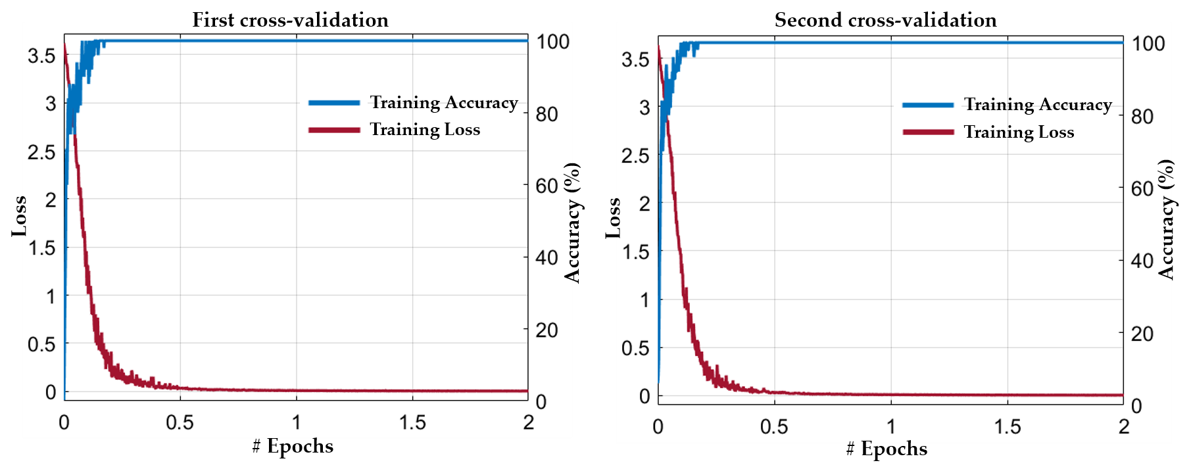


**Figure S8.** Average classification performance of our proposed network according to different numbers of successive frames (ie, *n* = 1,2,3, …,20) of the long short-term memory–based network in the second stage. mAP: mean average precision; mAR: mean average recall.


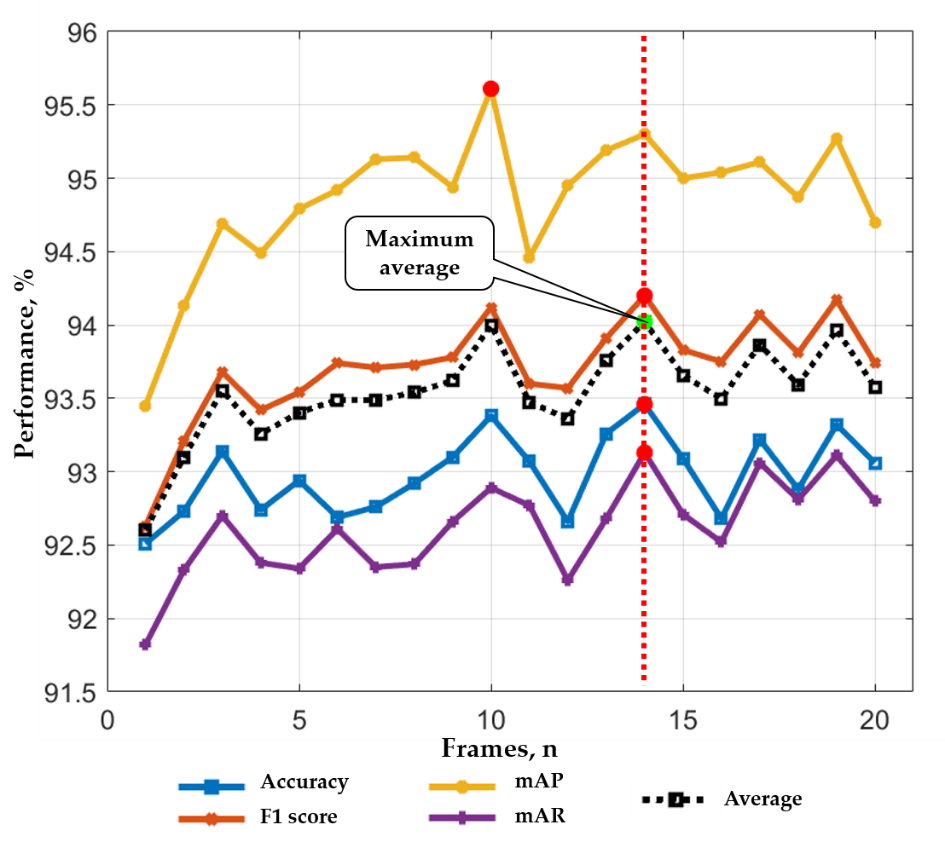


**Figure S9.** Average classification performance of our proposed method according to the different numbers of successive frames (ie, *n* = 1,2,3, …,150) in the testing phase. mAP: mean average precision; mAR: mean average recall.


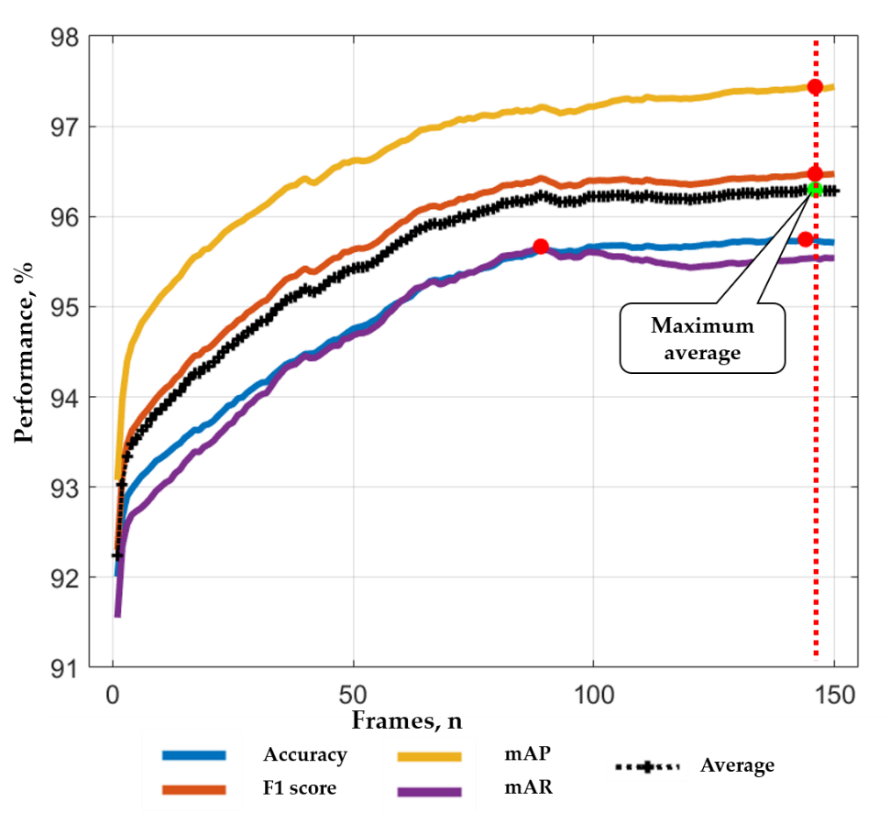


**Figure S10.** Principal component analysis–based performance for different numbers of eigenvectors (λ = 1,2,3, …600). mAP: mean average precision; mAR: mean average recall.


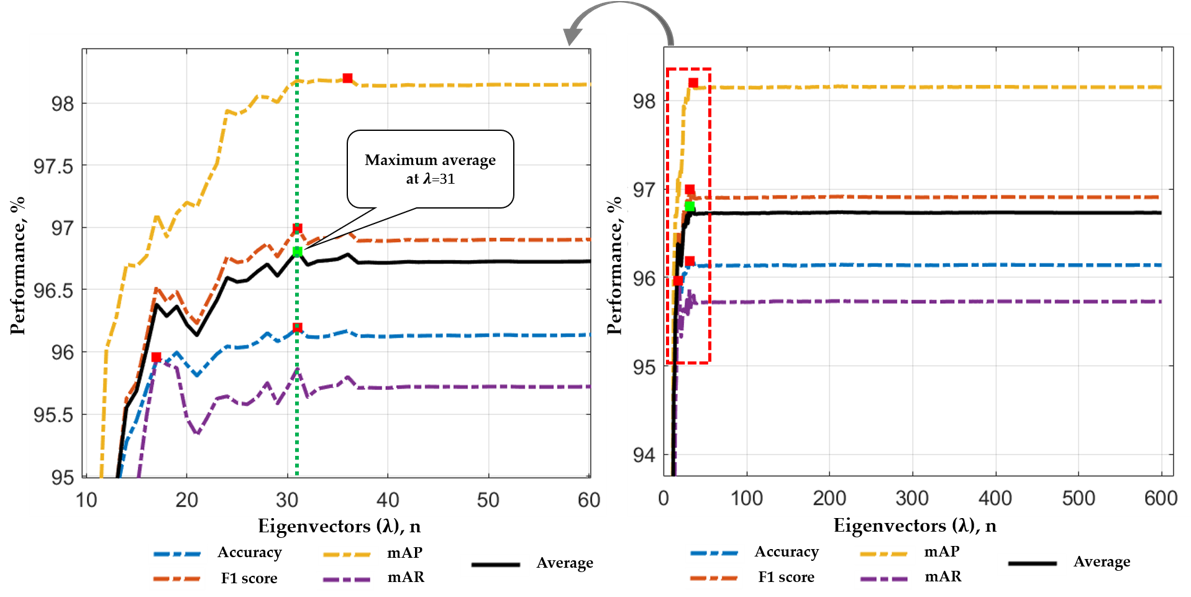


**Figure S11.** Sensitivity performance analysis results of our proposed model and the other baseline models for the average accuracy, F1 score, mAP, and mAR. CNN: convolutional neural network; LSTM: long short-term memory; mAP: mean average precision; mAR: mean average recall.


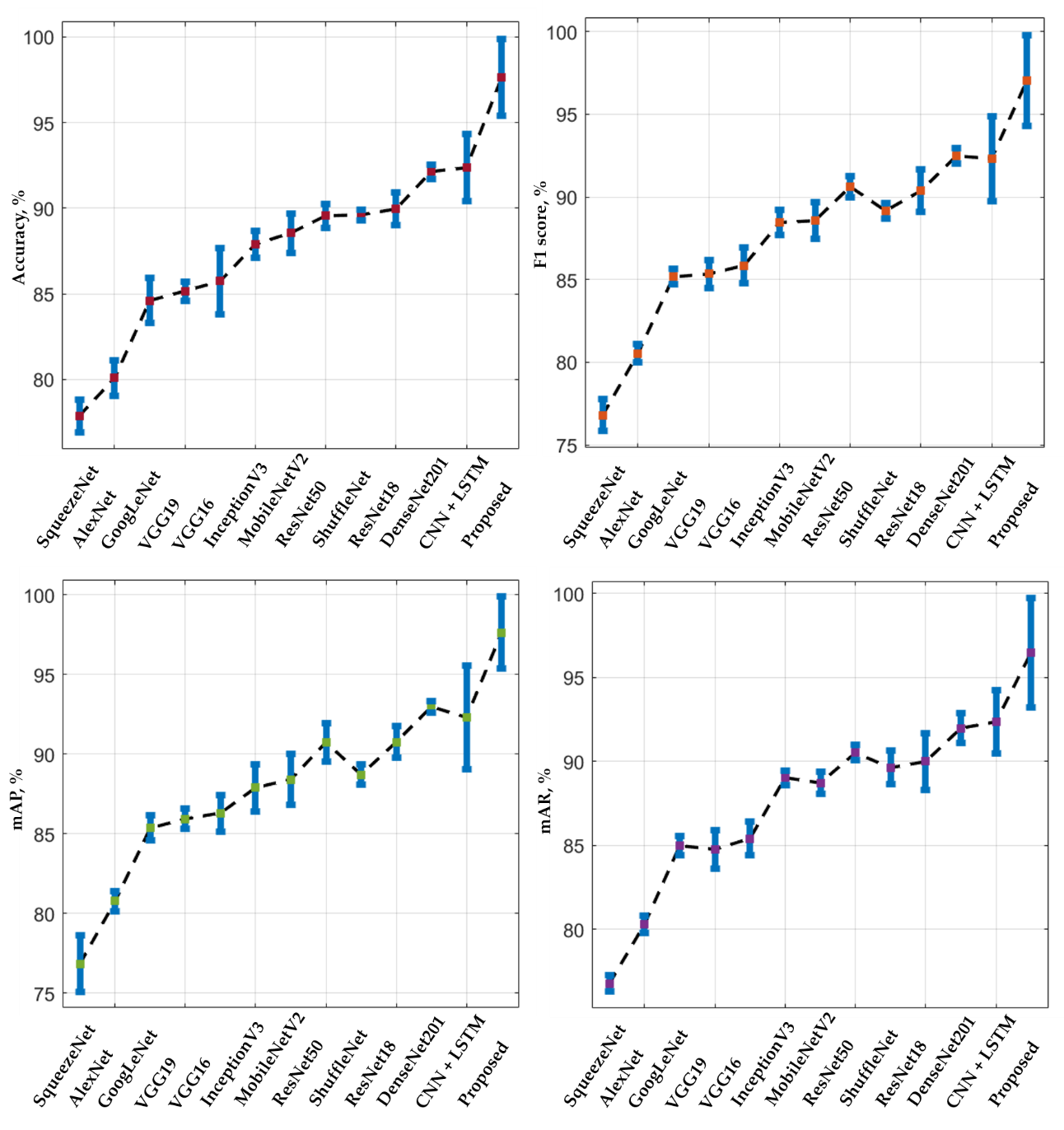

Supplement: Multimedia Appendix 1 [file jmir_v22i11e18563_app1.docx]
